# Regimen‐dependent glucocorticoid effects improve muscle performance without altering CNS physiology in *mdx* mice

**DOI:** 10.1113/JP291378

**Published:** 2026-05-28

**Authors:** Gretel S. Major, Jiayi Chen, Eva van den Berg, Deirdre L. Merry, Angus Lindsay

**Affiliations:** ^1^ School of Biological Sciences University of Canterbury Christchurch New Zealand; ^2^ Biomolecular Interaction Centre, School of Biological Sciences University of Canterbury Christchurch New Zealand; ^3^ Department of Medicine University of Otago Christchurch New Zealand; ^4^ Maurice Wilkins Centre for Molecular Biodiscovery Auckland New Zealand

**Keywords:** adrenal atrophy, behaviour, Duchenne muscular dystrophy, *in vivo* torque, prednisolone, stress hypersensitivity, vamorolone

## Abstract

**Abstract:**

Duchenne muscular dystrophy (DMD) is a multisystem disorder affecting striated muscle, metabolism and the central nervous system (CNS). Although glucocorticoids remain the standard therapy, muscle‐centric evaluations typically fail to capture how dosing regimen and compound selection affect CNS and metabolic phenotypes. Here, we compared daily and weekly dosing of prednisolone and vamorolone in juvenile *mdx* mice over 6 weeks to determine how these variables influence multisystem outcomes. Multiorgan efficacy and adverse effects were quantified across behavioural, endocrine, metabolic, cardiovascular and muscle domains using behavioural assays, *in vivo* and functional muscle testing, haemodynamic evaluation and histopathology. Daily glucocorticoid dosing failed to improve muscle function or strength, whereas weekly vamorolone produced the most robust improvements in functional and *in vivo* muscle strength. Daily prednisolone reduced circulating creatine kinase levels, but this biochemical change did not translate into enhanced muscle function outcomes. Daily regimens also induced severe adrenal cortical atrophy, yet these endocrine alterations were dissociated from CNS stress and anxiety responses, which remained unchanged by treatment. In addition, daily dosing caused pronounced systemic metabolic consequences, whereas weekly regimens substantially attenuated these effects, identifying dosing frequency as a key determinant of safety. Together, these findings demonstrate that glucocorticoid regimen selection fundamentally reshapes the efficacy *versus* adverse effect profile and underscores the value of integrated multiorgan evaluation in DMD. This work highlights the need to expand therapeutic assessments beyond muscle pathology and raises new questions about how glucocorticoid signalling differentially engages peripheral and central physiological systems.

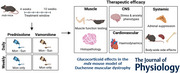

**Key points:**

- Duchenne muscular dystrophy affects multiple physiological systems, yet current glucocorticoid assessments rarely capture the CNS and metabolic effects of dosing regimens.
- Weekly vamorolone dosing improved muscle strength in *mdx* mice, while daily prednisolone failed to improve performance despite lowering plasma creatine kinase.
- Stress‐ and anxiety‐related behaviours remained unchanged, despite adrenal atrophy revealing a disconnect between peripheral endocrine disruption and CNS phenotypes.
- Daily glucocorticoid regimens triggered endocrine and metabolic side effects, whereas weekly dosing reduced these effects.
- Prednisolone and vamorolone produced broadly similar systemic side‐effect profiles, but dosing frequency was the primary determinant of their severity.

## Graphical abstract

**Abstract figure legend** Defining glucocorticoid efficacy in Duchenne muscular dystrophy requires assessment across muscle, central nervous system (CNS), cardiovascular and systemic domains. Here, juvenile mdx mice were treated over 6 weeks, comparing daily and weekly dosing of prednisolone and vamorolone. Weekly vamorolone improved muscle strength, whereas daily prednisolone lowered circulating creatine kinase with minimal functional benefit. Glucocorticoids did not alter stress and anxiety phenotypes in mdx mice, despite underlying differences from wild‐type animals. Daily prednisolone caused marked adrenal cortical atrophy, while vamorolone largely preserved adrenal structure. These findings demonstrate regimen‐dependent trade‐offs between muscle efficacy and systemic tolerance, underscoring the value of integrated, multi‐system approaches to optimise the therapeutic index.

## Introduction

Duchenne muscular dystrophy (DMD) while conventionally thought of as purely a severe muscle wasting condition, is becoming more commonly understood as a life‐limiting, multi‐system physiological disorder with central nervous system (CNS) involvement (Vaillend et al., 2025). Pathogenic variants within the *DMD* gene locus, which encodes the cytoskeletal protein dystrophin, result in reduced expression of multiple dystrophin isoforms across striated muscle and the CNS (Hoffman et al., 1987). Loss of dystrophin disrupts fundamental physiological processes including force transmission (Hughes et al., 2017), membrane stability (Goldstein & McNally, 2010) and excitation–contraction coupling in skeletal and cardiac muscle (Ullrich et al., 2009), culminating in progressive muscle weakness and cardiorespiratory insufficiency. In parallel, dystrophin deficiency within the CNS alters neural circuit function (Caudal et al., 2020; Vaillend & Chaussenot, 2017), stress responsivity (Maresh et al., 2023; Razzoli et al., 2020) and behavioural regulation (Saoudi et al., 2021), giving rise to complex neurocognitive and neurobehavioural comorbidities (e.g. attention deficit hyperactivity disorder, autism spectrum disorder features, lower IQ) which coexist with primary muscle pathology (Darmahkasih et al., 2020). Collectively, these manifestations reflect a system‐wide disturbance of physiological homeostasis rather than isolated tissue pathology, necessitating evaluation of therapeutic efficiency across multiple physiological domains, including skeletal muscle, the CNS and the cardiovascular system.

Glucocorticoids (GCs), including prednisolone, remain the only standard‐of‐care therapy for treating DMD, slowing disease progression and prolonging ambulation (Bello et al., 2015). GCs are proposed to delay DMD progression predominantly through anti‐inflammatory mechanisms that attenuate muscle degeneration (Angelini & Peterle, 2012). However, GC receptor activation exerts broad physiological effects across multiple organ systems, so chronic exposure is fraught with complexity and systemic burden, including impaired growth, osteoporosis, obesity, metabolic dysregulation, behavioural changes and suppression of the hypothalamic–pituitary–adrenal (HPA) axis (Handberg et al., 2022). These adverse effects reflect dose‐ and regimen‐dependent disruption of endocrine, musculoskeletal, cardiovascular and neurobehavioural physiology which substantially compromises patient quality of life. Clinical evidence suggests that intermittent GC dosing may preserve or enhance muscle function relative to daily administration (Bello et al., 2015; Connolly et al., 2002), with supporting preclinical studies at lower GC doses (Quattrocelli et al., 2017). Moreover, newer dissociative steroids such as vamorolone demonstrate reduced systemic side effects while retaining anti‐inflammatory efficacy (Heier et al., 2013), yet their impact on integrated physiological function, particularly cardiac haemodynamics and CNS‐related outcomes, remains incompletely characterised, and they have not been evaluated under intermittent dosing regimens.

Given that neurological manifestations occur in approximately half of individuals with DMD (Darmahkasih et al., 2020) and are associated with poorer clinical outcomes (Mochizuki et al., 2008), it is critical to understand how GC compounds and dosing regimens affect CNS functional outcomes within an integrated framework that also considers muscle structure and function, cardiovascular physiology and systemic metabolism. This 6 week study directly compared daily and weekly prednisolone (5 mg/kg/day) and vamorolone (30 mg/kg/day) in the juvenile *mdx* mouse model of DMD during rapid growth and active pathology, using micropipette‐guided dosing to minimise stress‐axis activation and preserve physiological accuracy (Ferreira‐Duarte et al., 2024; Scarborough et al., 2020). Therapeutic efficacy was assessed across skeletal muscle, cardiac and CNS phenotypes, alongside stress‐axis and endocrine side effects. By integrating multi‐organ physiological endpoints with multivariate analyses, this study defined how GC regimen and compound selection shape whole‐body physiological function in dystrophin deficiency, highlighting the importance of incorporating cardiovascular and neurophysiological measures into preclinical therapeutic evaluation for DMD.

## Materials and methods

### Ethical approval

Ethical approval for the research protocol and all procedures was obtained from the University of Otago Animal Ethics Committee (AUP‐24‐122). All procedures were performed in accordance with the Animal Welfare Act and the ARRIVE guidelines for animal experimentation (du Sert et al., 2020).

### Animal breeding and husbandry

Dystrophin‐negative C57BL/10ScSn‐*Dmd^mdx^*/J (*mdx*) and dystrophin‐positive C57BL/10ScSnJ (WT) mice were bred in‐house at the Christchurch Animal Research Area (AUP‐23‐115) from stock sourced from Jackson laboratories (Bar Harbor, ME, USA) on a 12 h light/12 h dark cycle, at 20–25°C and 40% humidity. A total of *n* = 42 mice were used in this study. Pups were weaned at 3 weeks of age and randomly assigned into cages of 3–4 mice based on treatment group. Body mass was measured every 2 days, and food consumption was recorded weekly for the duration of the study.

### Treatment protocol

**Figure 1:**
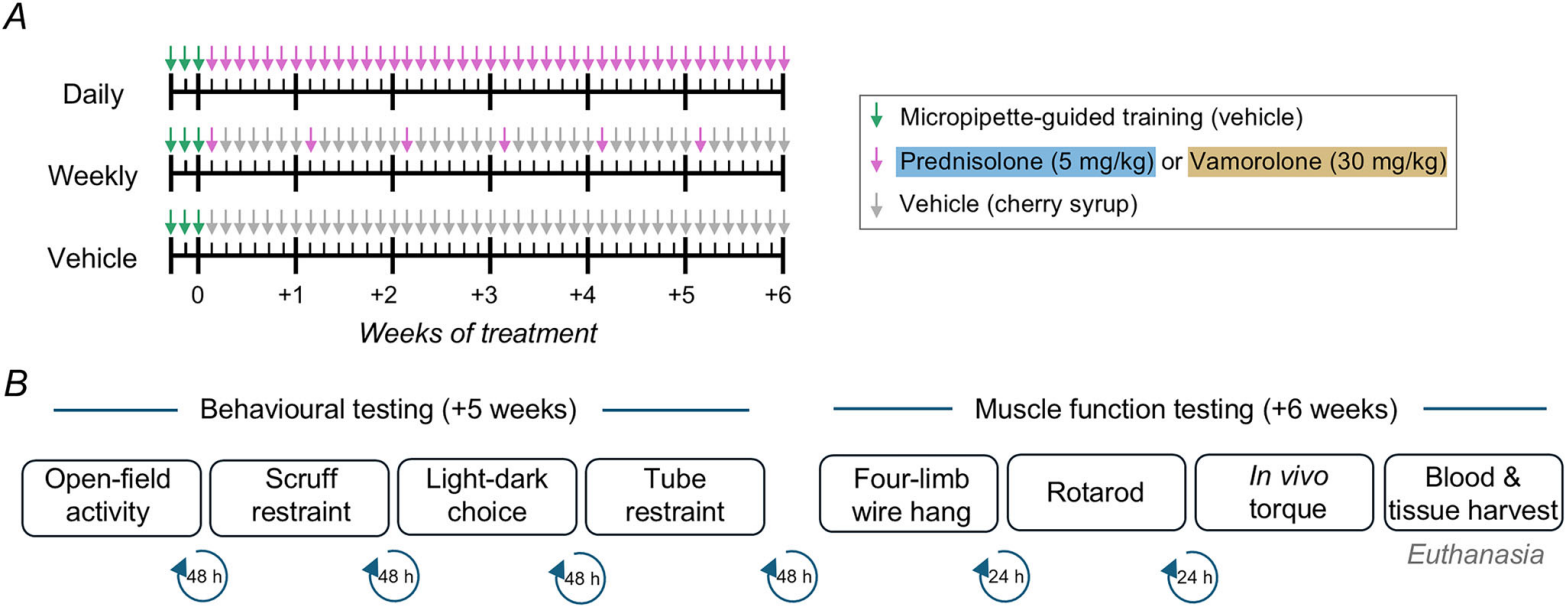
Overview of the treatment regimen and functional assessment timeline *A*, treatment schematic: daily or weekly oral dosing of prednisolone (5 mg/kg/day) or vamorolone (30 mg/kg/day) was administered over a 6 week treatment period, alongside vehicle‐treated *mdx* and wild‐type controls. All treatments were delivered via voluntary micropipette‐guided administration following a 3 day acclimation period to minimise stress‐axis activation. Weekly groups received the full dose once per week with vehicle on remaining days, whereas daily groups received compound‐matched doses each morning. Mice continued on their assigned treatment regimen until euthanasia. *B*, behavioural and muscle‐function assessment timeline: testing batteries were conducted after 5 and 6 weeks of treatment. Behavioural assessments were spaced at 48 h intervals, while muscle‐function assessments were spaced at 24 h intervals. Immediately following *in vivo* torque assessments, mice were killed and blood and tissues were collected.

*Mdx* mice were treated orally with GCs in daily or weekly regimens for 6 weeks from 4 to 10 weeks of age, capturing the period where muscle degeneration peaks and aligning with treatment windows in previous studies (Fig. 1) (Kourakis et al., 2024; Timpani et al., 2023; Ziemba et al., 2021). Six experimental groups were evaluated: five *mdx* treatment groups – (1) cherry syrup vehicle, (2) 5 mg/kg prednisolone daily, (3) 5 mg/kg prednisolone weekly, (4) 30 mg/kg vamorolone daily and (5) 30 mg/kg vamorolone weekly – and a sixth group of WT mice receiving the cherry syrup vehicle as a healthy control. Group sizes were *n* = 7 mice per group and the same animals were used across longitudinal, behavioural, *in vivo* functional, biochemical and histological analyses. For brain mass and behavioural outcomes, final analyses were performed with *n*  =  6 mice in the *mdx* vehicle control group following post‐mortem identification of hydrocephalus at endpoint dissection.

Animals were weighed at 7–8 a.m. every 2 days, and individual treatments were prepared daily in cherry syrup vehicle relative to body mass to give a final dosage of either 5 mg/kg/day prednisolone or 30 mg/kg/day vamorolone. Mice receiving GC treatments on once weekly regimens received the cherry syrup vehicle on the remaining 6 days. The amount of cherry syrup vehicle administered was standardised across all treatment groups to achieve 1 µL/g body mass/day (Heier et al., 2013, 2019; Liu et al., 2024; McCormack et al., 2023). The oral prednisolone dose (5 mg/kg/day) was selected based on established dosing paradigms shown to modify muscle pathology and function in *mdx* mice (Kourakis et al., 2024; Liu et al., 2024). The intermittent glucocorticoid regimen was informed by prior studies demonstrating beneficial effects using intraperitoneal administration of prednisolone at 1 mg/kg/day (Quattrocelli et al., 2017, 2019). Intermittent dosing has not previously been examined for vamorolone; therefore, the 30 mg/kg/day oral dose was chosen based on studies demonstrating efficacy with daily administration in dystrophic mice (Heier et al., 2013, 2019; Liu et al., 2024; McCormack et al., 2023).

Given that *mdx* mice demonstrate stress hypersensitivity in response to a scruff restraint (Gharibi et al., 2024; Lindsay & Russell, 2023) and that a primary aim of this study was to determine the effects of GC treatment regimens on behavioural phenotypes in DMD, all treatments were delivered by voluntary micropipette‐guided administration. This administration route has been shown to produce similar pharmacokinetic profiles to oral gavage with reduced cortisol production (Ferreira‐Duarte et al., 2024; Scarborough et al., 2020). All mice were trained to drink the cherry syrup vehicle from a 200 µL pipette for 3 days prior to the beginning of the treatment regimen by positioning a pipette tip containing the cherry syrup vehicle close to the mouth until the mouse drank. All mice were successfully trained after 2 days and there were no instances of failed drug delivery throughout the study.

### Blood glucose

To determine the effects of the treatment regimens on systemic glucose regulation, blood glucose concentrations were measured every 2 weeks. At week 4, mice were fasted for 5 h (from ∼8 a.m. to ∼1 p.m.) and then blood glucose was measured from a tail nick using a blood glucometer (OneTouch Select Plus). For endpoint assessments (after 6 weeks of treatment), measurements were taken in an unfasted state directly prior to termination.

### Behavioural testing battery

The behavioural testing battery was employed after 5 weeks of treatment (Fig. 1). To minimise cumulative burden and interaction between behavioural tests, the three tests were conducted 48 h apart. The order of testing was as follows: open‐field activity, scruff restraint stress test, light–dark choice and then tube‐restraint stress test with haemodynamic evaluation.

#### Open‐field activity

Total distance travelled in the open‐field was measured over 10 min in a plexiglass testing chamber (40 × 40 × 40 cm length/width/height, Omnitech Electronics, Ohio, USA) in a room with homogeneous dim illumination (∼50 Lx) using Fusion software (AccuScan, Omnitech Electronics).

#### Scruff restraint stress test

To evaluate stress hypersensitivity, physical activity was measured for 5 min following a scruff restraint stressor, as described previously (Lindsay, Trewin et al., 2021; Razzoli et al., 2020; Sekiguchi et al., 2009). Mice were grasped by the nape between the thumb and index finger, securing the tail between the fourth and fifth finger, and placed in the supine position for 30 s. Following the scruff restraint the mice were immediately placed in a sterile open‐field activity monitoring chamber (∼20 ×  20 ×  40 cm H; Omnitech Electronics). Physical activity was quantified as total movement time over 5 min and reported as both total time and percentage freezing in 1 min bins (AccuScan, Omnitech Electronics).

#### Light–dark choice

Anxiety was measured in a light–dark choice apparatus with monitoring over 5 min. The testing apparatus consisted of a plexiglass box with a brightly lit compartment (40 ×  20 × 30 cm; illumination: 600 Lx) connected by an animal entry opening with a trapdoor (10 × 4 cm) to a dark compartment (40 × 15 × 20 cm; illumination: <10 Lx) (Omnitech Electronics). Each mouse was placed in the dark compartment for 10 s before the trapdoor was opened and mice were allowed to freely explore the apparatus for 5 min. Number of entries and total time spent in the lit compartment were recorded using Fusion software (AccuScan, Omnitech Electronics).

#### Tube‐restraint stress test with cardiovascular haemodynamic measures

The haemodynamic response to stress was measured using a 5 min tube‐restraint stressor with concurrent evaluation of cardiovascular haemodynamic parameters. Mice were placed in the CODA^®^ non‐invasive blood pressure system using a plexiglass tube mouse holder suitable for the mouse body mass (Kent Scientific, Torrington, CT, USA) for 5 min, as previously described (Gharibi et al., 2025). The mouse holders were placed on a 37°C warming platform, and tail cuffs were positioned to expose the tip of the tail. Blood pressure was recorded every 30 s for a total of 10 measurements using volume pressure recording sensor technology and CODA software. Mean arterial pressure, heart rate, tail blood volume and shock index (the ratio of maximum heart rate to lowest systolic blood pressure, indicating hypovolaemic stress) were recorded. At the completion of the haemodynamic measures, mice were placed into a sterile physical activity monitoring chamber for 5 min to assess physical activity after the stressor (as described above).

### Muscle function testing battery

Functional muscle strength was tested after 6 weeks of treatment using the following testing battery: four‐limb wire hang, rotarod performance, and *in vivo* strength of the anterior and posterior crural muscles (24 h between each test; Fig. 1).

#### Four‐limb wire hang

The four‐limb wire hang test assessed whole body strength by measuring the ability of mice to maintain grip on an inverted wire grid mesh (∼1 × 1 cm mesh) (Treat‐NMD protocol DMD_M.2.1.004). The hanging time from mesh inversion was recorded and the test was repeated over three trials for each mouse (15 min between trials). The holding impulse was calculated as body mass multiplied by absolute hang time for the longest trial. One mouse was excluded as it refused the test (hanging < 10 s on three repeated attempts).

#### Rotarod

A rotarod performance test assessed neuromotor coordination over three trials. One day before testing, mice underwent two acclimatisation sessions, 30 min apart, during which they ran for 1 min at 5 rpm before the speed was ramped up to 45 rpm. For the test, each trial started with a stabilisation period at 5 rpm, followed by an acceleration to 45 rpm over 30 s and maintenance at 45 rpm until the mouse fell from the rotarod (or 600 s was reached). Time to fall was recorded for each trial, with the longest trial used for data analysis.

#### *In vivo* muscle strength

Maximum isometric strength of the anterior and posterior crural muscles was assessed by stimulation of the common peroneal nerve and sciatic nerve, respectively, using percutaneously placed electrodes connected to a stimulator–force transducer apparatus (Aurora Scientific, Canada). Mice were maintained under anaesthesia (details below) throughout and peak isometric tetanic torque was measured by manipulating voltage at 200 Hz every min until a plateau was attained (within 0.01 mN.m). Maximal rate of contraction and maximal rate of relaxation were assessed using peak torque. Torque was analysed as specific torque, that is maximum tetanic torque normalised by the combined tibialis anterior (TA) and extensor digitorum longus (EDL) mass for the anterior crural muscles and the gastrocnemius and soleus of the posterior crural muscles.

### Anaesthesia and euthanasia

Mice were anaesthetised for *in vivo* skeletal muscle function assessment using isoflurane delivered in 95% oxygen. Anaesthesia was induced in an induction chamber (5% isoflurane) and maintained via a nose cone at 1–3% isoflurane for the duration of the experiment. Body temperature was maintained at 37°C using a thermostatically controlled heating pad. Depth of anaesthesia was assessed and monitored throughout by absence of the pedal withdrawal reflex. Upon completion of the experimental protocol, mice remained under deep isoflurane anaesthesia and terminal blood was collected by intracardiac puncture. Animals were then humanely killed by cervical dislocation while fully unconscious.

### Tissue harvest

After end‐point *in vivo* muscle function testing and blood collection, the organs (brain, adrenal glands, spleen) and striated muscles (diaphragm, TA, EDL, soleus, gastrocnemius and quadriceps) were excised, weighed and snap‐frozen in liquid nitrogen for further assays (unless stated otherwise below).

### Creatine kinase

Muscle damage markers were assessed by determination of plasma creatine kinase (CK) levels. Blood was collected via terminal cardiac puncture into lithium heparin microtubes. Plasma was derived by centrifugation (3000 *g*, 5 min, 4°C) and was stored at −80°C until assayed. CK levels were quantified spectrophotometrically using a CK‐NAC kit as per the manufacturer's instructions (CK8313, Randox Laboratories, Kearneysville, WV, USA).

### Histopathology and immunohistochemistry

The right TA (opposite leg used for *in vivo* strength testing) and right hemisphere of the diaphragm were coated in OCT (TissueTek) and snap‐frozen in liquid nitrogen‐cooled isopentane. Muscle samples were thawed to −20°C and cryosectioned at 10 µm. To visualise tissue structure, diaphragm sections were fixed in methanol and stained with haematoxylin and eosin. For quantification of myofibres, mid‐belly sections of the TA were fixed in −20°C acetone for 5 min, blocked in 5% BSA/PBS, and counterstained with laminin (1:500; Sigma‐Aldrich L9393) for 2 h at room temperature as previously described (Devananthan et al., 2026). Sections were incubated with anti‐Rabbit Alexa Fluor 488 (1:500; ThermoFisher Scientific A11008, Waltham, MA, USA) for 1 h at room temperature and mounted in ProLong Golf Antifade with DAPI (ThermoFisher Scientific). Images were acquired on a Zeiss AxioImager Z1 fluorescence microscope and stitched together with Zen software (Zeiss, Oberkochen, Germany). SMASH software was used to quantify number myofibres, myofibre cross‐sectional area (CSA) and centronucleated fibres (Smith & Barton, 2014). Whole muscle CSA was measured manually using ImageJ.

The right adrenal gland was immediately fixed in 4% paraformaldehyde for 30 min at room temperature, coated in OCT and snap‐frozen in liquid nitrogen‐cooled isopentane. Adrenal glands were thawed to −20°C and cryosectioned at 4 µm, before staining with haematoxylin and eosin. Images were acquired on a Zeiss AxioImager Z1 microscope and CSA, cortex area and medulla area were measured manually using ImageJ.

### Hydroxyproline assay

Muscle fibrosis was assessed by measuring hydroxyproline content of the left hemisphere of the diaphragm according to the manufacturer's instructions (AB222941, Abcam, Cambridge, MA, USA).

### Statistics

All data are reported as mean ± SD unless otherwise stated. Data were analysed using R and visualised using GraphPad Prism 9. A permutational multivariate analysis of variance (PERMANOVA) was conducted using the Euclidean method to assess overall group differences in multivariate composition across muscle parameters (time to fall during the rotarod test, holding impulse during the four‐limb wire hang, maximum isometric torque, CK levels, hydroxyproline content, centronucleated fibres), behavioural parameters (total distance travelled in the open‐field, total movement time following a scruff restraint, total movement time following a tube restraint, time in the lit compartment of light dark choice apparatus, brain mass) or cardiac parameters (blood pressure, heart rate, blood flow, heart mass). Heart rate measurements could not be obtained for six mice (four *mdx* vehicle controls, one weekly vamorolone and one weekly prednisolone), resulting in missing data for this variable only. In addition, one mouse in the daily vamorolone group refused the four‑limb wire hang test and was excluded from that specific analysis. Missing data were imputed solely for the purpose of multivariate PERMANOVA following standardisation of variables. No imputation was applied to any univariate analyses. Excluding the mice with missing values entirely yielded comparable PERMANOVA results.

For individual outcome measures, group comparisons were performed using one‐way ANOVA for single‐factor designs, two‐way ANOVA for repeated‐measures behavioural outcomes (binned freezing behaviour) or Kruskal–Wallis test when normality assumption was not met and could not be corrected (light–dark apparatus), with a *P* value of <0.05 considered statistically significant. Where relevant, *post hoc* Tukey's HSD tests were used for multiple comparisons following ANOVA. Details of the statistical test used and main effects *P* values for all outcome measures are detailed in Table A1. WT mice were included in all statistical tests; however, results are described with emphasis on treatment‐related differences within *mdx* mice and comparisons indicating restoration towards WT levels. One mouse from the *mdx* vehicle control group was removed from all the CNS analysis retrospectively, when hydrocephalus was identified.

## Results

### Prednisolone and vamorolone generate distinct multivariate profiles of muscle functional, contractile and structural physiology

**Figure 2:**
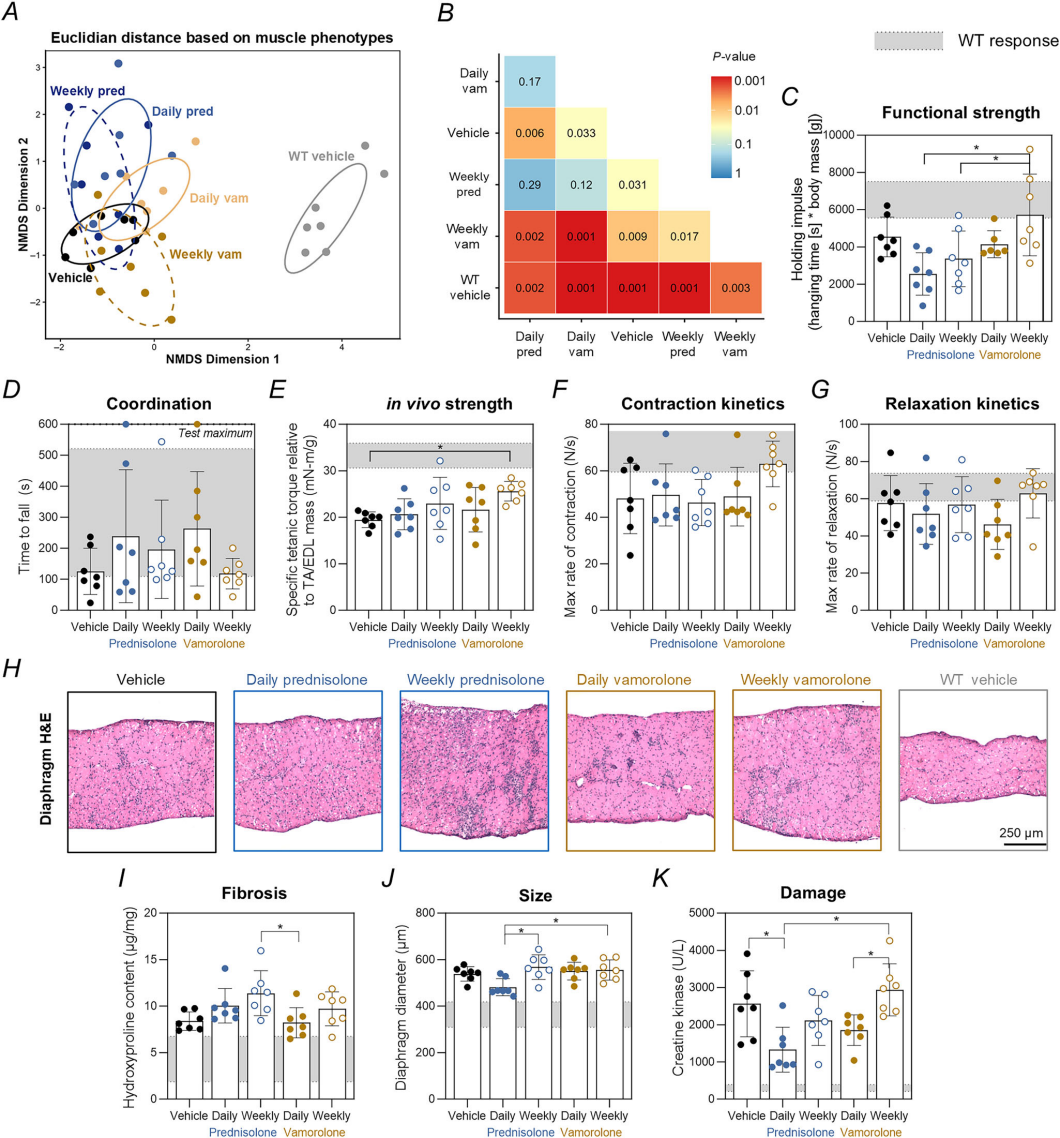
Prednisolone and vamorolone produce divergent profiles of muscle function, contractility and tissue integrity, reflecting drug‐specific physiological effects *A*, on‐metric multidimensional scaling (NMDS) ordination based on Euclidean distance illustrating multivariate skeletal muscle physiological profiles across experimental groups, including prednisolone (blue) and vamorolone (yellow) treatments, and WT (black) and *mdx* (grey) vehicle controls. Each point represents an individual animal, and proximity reflects similarity in integrated physiological profiles. *B*, heat map of pairwise *P*‐values for between‐group multivariate comparisons across all experimental groups. *C*, four‐limb wire hang test assessing functional strength, expressed as holding impulse. *D*, rotarod performance assessing motor coordination, expressed as time to fall. *E*, *in vivo* muscle strength expressed as specific tetanic torque normalised to tibialis anterior (TA) and extensor digitorum longus (EDL) muscle mass. *F*, contraction kinetics of the anterior crural muscles expressed as maximal rate of torque development. *G*, relaxation kinetics of the anterior crural muscles expressed as maximal rate of torque relaxation. *H*, representative haematoxylin and eosin (H&E)‐stained sections of diaphragm muscle (scale bar = 250 µm). *I*, diaphragm fibrosis quantified by hydroxyproline content. *J*, diaphragm muscle size assessed by muscle diameter. *K*, plasma creatine kinase concentration as a marker of muscle damage. Multivariate group differences were assessed using Euclidean distance‐based PERMANOVA on z‐score standardised data, with dispersion tested by betadisper. Significant global effects were followed by pairwise multivariate comparisons, and outcome‐specific differences were assessed using univariate ANOVA with appropriate correction for multiple testing; *P* < 0.05. WT responses are shown in grey shading as ±SD. All other data are presented as mean ± SD; *n* = 7 mice per group.

To capture treatment‐dependent effects across muscle contractile, structural and functional parameters, we performed non‐metric multidimensional scaling (NMDS) on a combined dataset integrating a subset of physiological outcome measures (four‐limb wire hang, rotarod performance, *in vivo* torque testing, plasma CK levels, muscle hydroxyproline content and the proportion of centrally nucleated fibres; Fig. 2*A*, *B*). These variables were chosen from the broader dystrophic analysis panel because they reflect complementary aspects of muscle pathology and did not correlate with one another, ensuring that each provided independent physiological information within the integrated analysis. Both GCs, independent of dosing regimen, altered global muscle physiology relative to *mdx* vehicle controls (daily prednisolone: *P* = 0.006; weekly prednisolone: *P* = 0.031; daily vamorolone: *P *= 0.033; weekly vamorolone: *P* = 0.009), with no distinction between daily and weekly prednisolone (*P *= 0.29), or between daily prednisolone and daily vamorolone (Fig. 2*A*, *B*; *P *= 0.17). Daily administration of prednisolone produced the largest physiological shift (*P* = 0.006), followed closely by weekly vamorolone (Fig. 2*A*, *B*; *P* = 0.009).

**Figure A1:**
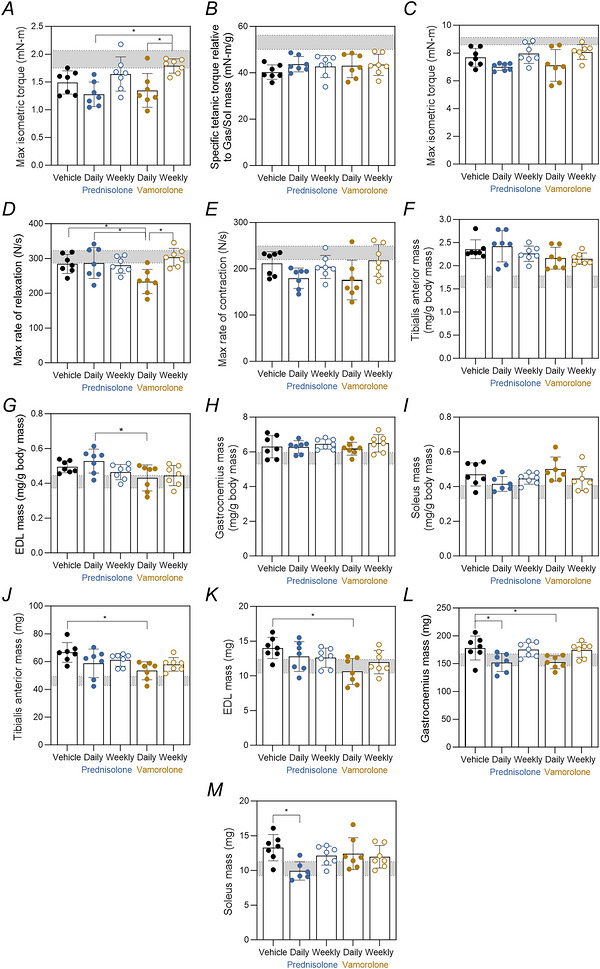
Evaluation of absolute isometric strength of the anterior crural muscles, posterior leg muscle isometric strength, contractile properties and muscle mass *A*, absolute *in vivo* isometric muscle strength of the anterior crural muscles. *B*, *in vivo* isometric muscle strength expressed as specific tetanic torque normalised to gastrocnemius (gas) and soleus (sol) muscle mass. *C*, absolute *in vivo* isometric muscle strength of the posterior leg muscles. *D*, contraction kinetics of posterior leg muscles expressed as maximal rate of torque development. *E*, relaxation kinetics of posterior leg muscles expressed as maximal rate of torque relaxation. *F*, tibialis anterior mass expressed relative to body mass. *G*, EDL muscle mass expressed relative to body mass. *H*, gastrocnemius mass expressed relative to body mass. *I*, soleus mass expressed relative to body mass. *J*, absolute tibialis anterior mass. *K*, absolute EDL mass. *L*, absolute gastrocnemius mass. *M*, absolute soleus mass. Group differences were assessed using univariate ANOVA; *P* < 0.05. WT responses are shown in grey shading as ± SD. All other data are presented as mean ± SD; *n* = 7 mice per group.

When evaluating individual physiological outcomes, weekly vamorolone improved functional strength, measured by four‐limb holding impulse, relative to both daily (*P* = 0.001) and weekly prednisolone (*P* = 0.026), and was the only group not different from WT vehicle controls (Fig. 2*C*; *P* = 0.87). Rotarod coordination was unaffected by any treatment relative to *mdx* vehicle controls (daily prednisolone: *P* = 0.82; weekly prednisolone: *P* = 0.84; daily vamorolone: *P *= 0.50; weekly vamorolone: *P* = 1.00), while only weekly vamorolone enhanced *in vivo* strength of the anterior crural muscles relative to vehicle‐treated *mdx* mice (*P* = 0.036), without altering contraction (*P* = 0.20) or relaxation kinetics (Fig. 2*D*–*G*; Fig. A1; *P* = 0.98). No treatment improved the strength (daily prednisolone: *P* = 0.62; weekly prednisolone: *P* = 0.88; daily vamorolone: *P *= 0.83; weekly vamorolone: *P* = 0.71) or contraction rates (daily prednisolone: *P* = 0.34; weekly prednisolone: *P* = 1.00; daily vamorolone: *P *= 0.22; weekly vamorolone: *P* = 1.00) of the posterior crural muscles relative to *mdx* vehicle controls, but daily vamorolone slowed relaxation relative to daily prednisolone (*P* = 0.024), weekly vamorolone (*P* = 0.002) and vehicle‐treated *mdx* controls (Fig. A1*B*–*E*; *P* = 0.038).

Structurally, no treatment reduced diaphragm fibrosis, as measured by hydroxyproline content, although weekly prednisolone treatment led to greater fibrosis relative to daily vamorolone‐treated mice (Fig. 2*H*, *I*; *P* = 0.042). Daily prednisolone decreased diaphragm thickness relative to weekly dosing of both prednisolone (*P* = 0.009) and vamorolone (Fig. 2*H*, *J*; *P* = 0.036). However, the normalised mass (muscle weight relative to body weight) of the EDL (daily prednisolone: *P* = 0.82; weekly prednisolone: *P* = 0.27; daily vamorolone: *P *= 0.27; weekly vamorolone: *P* = 0.50), TA (daily prednisolone: *P* = 0.99; weekly prednisolone: *P* = 0.96; daily vamorolone: *P *= 0.53; weekly vamorolone: *P* = 0.47), soleus (daily prednisolone: *P* = 0.50; weekly prednisolone: *P* = 0.97; daily vamorolone: *P *= 0.90; weekly vamorolone: *P* = 0.96) or gastrocnemius (daily prednisolone: *P* = 1.00; weekly prednisolone: *P* = 0.98; daily vamorolone: *P *= 1.00; weekly vamorolone: *P* = 0.95) did not differ among treatment groups relative to *mdx* vehicle controls (Fig. A1*F*–*M*).

Muscle damage, as indicated by plasma CK, was reduced only by daily prednisolone relative to vehicle controls (*P* = 0.008). Weekly vamorolone‐treated mice had higher CK than both daily prednisolone‐ (*P* = 0.0003) and daily vamorolone‐treated mice (*P* = 0.024), demonstrating that daily GC administration is required to mitigate muscle damage, and that daily prednisolone was the only regimen associated with a significant reduction in circulating CK relative to *mdx* vehicle controls (Fig. 2*K*).

### Daily prednisolone alters muscle fibre organisation relative to vamorolone

**Figure 3:**
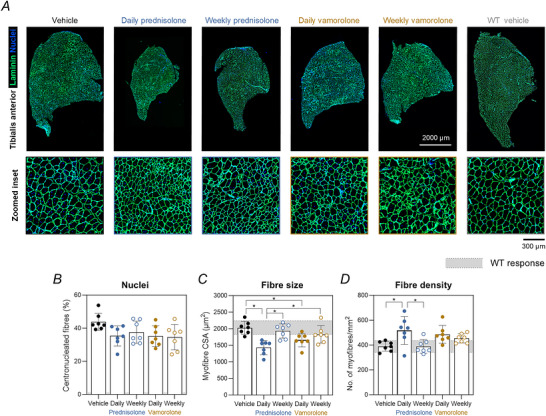
Daily prednisolone enhances fibre number and reduces fibre size, effects less pronounced with vamorolone *A*, representative laminin (green) and nuclei (blue) immunofluorescence images of tibialis anterior (TA) muscle, showing fibre architecture and myonuclear positioning (scale bar = 250 µm). *B*, percentage of centrally nucleated fibres. *C*, myofibre cross‐sectional area (CSA). *D*, fibre density (fibres/mm^2^). Group differences were assessed using univariate ANOVA; *P* < 0.05. WT responses are shown in grey shading as ± SD. All other data are presented as mean ± SD; *n* = 7 mice per group.

**Figure A2:**
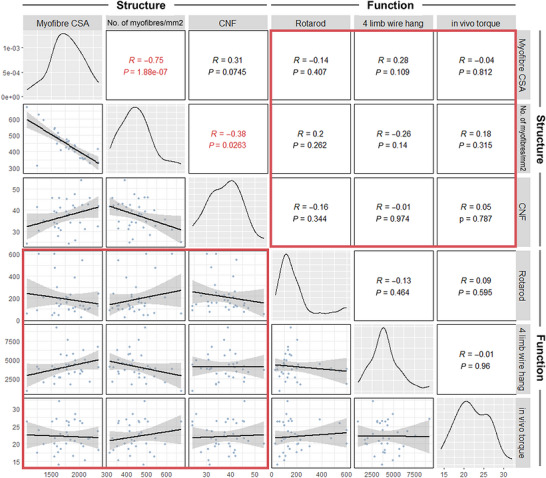
Relationship between muscle structural and functional parameters in *mdx* muscle Pairwise scatterplots showing associations between architectural muscle fibre characteristics (myofibre cross‐sectional area, fibre number density and proportion of centronucleated fibres) and functional outcomes (rotarod performance, four‐limb wire hang and *in vivo* torque) in *mdx* mice. Lower panels show individual data points with linear regression fits and 95% confidence intervals; upper panels show Pearson correlation coefficients (*R*) and corresponding *P*‐values.

To further investigate how GC treatment shapes skeletal muscle architecture that underlies contractile performance, we examined muscle fibre organisation. No treatment altered the percentage of centrally nucleated fibres relative to *mdx* vehicle controls (daily prednisolone: *P* = 0.095; weekly prednisolone: *P* = 0.37; daily vamorolone: *P *= 0.091; weekly vamorolone: *P* = 0.055), indicating comparable levels of ongoing regeneration across groups (Fig. 3*A*, *B*). However, both daily prednisolone (*P* = 0.0001) and daily vamorolone (*P* = 0.033) reduced myofibre CSA relative to *mdx* vehicle controls (Fig. 3*A*, *C*). In prednisolone‐treated mice, fibre size was also influenced by dosing frequency (*P* = 0.002), reflecting reduced fibre CSA with daily dosing (Fig. 3*A*, *C*). In contrast, vamorolone‐treated mice showed consistent fibre sizes across both dosing regimens (Fig. 3*A*, *C*; *P* = 0.68). Daily prednisolone treatment further increased fibre number per unit area relative to vehicle controls (*P* = 0.011) and weekly prednisolone treatment (*P* = 0.010), an effect not observed with daily (*P* = 0.092) or weekly (*P* = 0.47) vamorolone treatment (Fig. 3*A*, *D*). Importantly, structural parameters measured in the TA, including fibre size, density and central nucleation, did not correlate with anterior crural *in vivo* torque or whole‑body functional performance measures (rotarod performance and four‐limb wire holding impulse) in *mdx* mice (Fig. A2). These findings indicate that tibialis anterior muscle architecture alone is insufficient to predict functional outcomes across anatomically distinct and multi‐muscle tasks.

### Glucocorticoid treatment does not alter brain structure, anxiety or stress‐axis physiology

**Figure 4:**
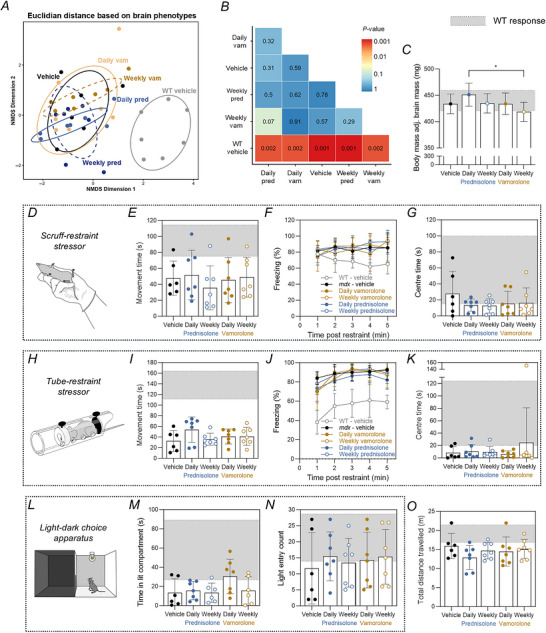
Prednisolone and vamorolone did not alter stress‐ oranxiety‐related behavioural outcomes *A*, non‐metric multidimensional scaling (NMDS) ordination based on Euclidean distance illustrating multivariate brain and behavioural profiles across experimental groups, including prednisolone (blue) and vamorolone (yellow) treatments, and WT (black) and *mdx* (grey) vehicle controls. Each point represents an individual animal, and proximity reflects similarity in integrated functional profiles. *B*, heat map of pairwise *P*‐values for between‐group multivariate comparisons across all experimental groups. *C*, body‐weight‐adjusted brain mass across treatment groups. Data are estimated marginal means (±SD) derived from an ANCOVA model with body weight included as a covariate. *Scruff restraint stress: D*, schematic of the scruff restraint stress paradigm; *E*, movement time following scruff restraint stress; *F*, percentage of time spent freezing in 1 min bins; *G*, time spent in the centre of the cage. *Tube restraint stress: H*, schematic of the tube restraint stress paradigm; *I*, movement time following tube‐restraint stress; *J*, percentage of time freezing in 1 min bins; *K*, time spent in the centre of the cage. *Light–dark choice anxiety test: L*, schematic of the light–dark choice test; *M*, time spent in the lit compartment; *N*, number of entries into the lit compartment. *Open field behaviour: O*, total distance travelled over 10 min. Multivariate group differences were assessed using Euclidean distance‐based PERMANOVA on z‐score standardised data, with dispersion tested by betadisper. Significant global effects were followed by pairwise multivariate comparisons, and outcome‐specific differences were assessed using univariate ANOVA, two‐way ANOVA or Kruskall–Wallis test with appropriate correction for multiple testing; *P* < 0.05. WT responses are shown in grey shading as ± SD. All other data, except where otherwise specified, are presented as mean ± SD; *n* = 6–7 mice per group.

**Figure A3:**
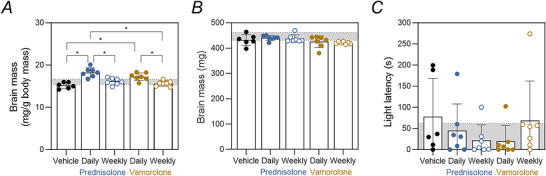
Assessment of brain size and anxiety‐related behaviour *A*, brain mass expressed relative to body mass. *B*, absolute brain mass. *Light–dark choice anxiety test: C*, latency to enter lit compartment. Group differences were assessed using univariate ANOVA or Kruskall–Wallis *H* test; *P* < 0.05. WT responses are shown in grey shading as ±SD. All other data are presented as mean ± SD; *n* = 7 mice per group.

To assess the impact of prednisolone and vamorolone on CNS physiology in DMD, we examined brain size along with behavioural outcomes related to anxiety and stress‐reactivity, characteristics of this DMD model. We then performed NMDS to integrate these CNS‐related parameters, including responses to scruff‐restraint, tube‐restraint, light–dark choice, open‐field and brain weight, into a single composite analysis. The NMDS revealed clear separation between vehicle‐treated WT and *mdx* mice (*P* = 0.004), reflecting genotype‐dependent differences in overall brain physiology, but no treatment groups diverged from *mdx* vehicle controls (Fig. 4*A*, *B*; daily prednisolone: *P* = 0.31; weekly prednisolone: *P* = 0.78; daily vamorolone: *P *= 0.59; weekly vamorolone: *P* = 0.57). Analysis of body mass‐adjusted brain mass showed no effect of treatment relative to vehicle controls (daily prednisolone: *P* = 0.61; weekly prednisolone: *P* = 1.00; daily vamorolone: *P *= 1.00; weekly vamorolone: *P* = 0.73), though daily prednisolone resulted in larger brain mass relative to weekly vamorolone (Fig. 4*C*; Fig. A3*A*, *B*; *P* = 0.040).

To investigate stress‐axis responsiveness, we applied mild (scruff restraint) and moderate (tube restraint) laboratory stressors and tracked movement patterns following the stressor (Fig. 4*D*, *H*). Neither paradigm produced treatment‐dependent differences in total movement time [*Scruff restraint* (daily prednisolone: *P* = 1.00; weekly prednisolone: *P* = 0.67; daily vamorolone: *P *= 1.00; weekly vamorolone: *P* = 1.00); *Tube restraint* (daily prednisolone: *P* = 0.55; weekly prednisolone: *P* = 0.99; daily vamorolone: *P *= 0.93; weekly vamorolone: *P* = 0.83)], binned freezing behaviour (*Scruff restraint*: *P* = 0.82; *Tube restraint*: *P* = 0.18), or centre exploration relative to *mdx* vehicle controls [Fig. 4*D*–*K*; *Scruff restraint* (daily prednisolone: *P* = 1.00; weekly prednisolone: *P* = 0.99; daily vamorolone: *P *= 0.99; weekly vamorolone: *P* = 1.00); *Tube restraint* (daily prednisolone: *P* = 0.99; weekly prednisolone: *P* = 0.97; daily vamorolone: *P *= 1.00; weekly vamorolone: *P* = 1.00)]. Importantly, despite previous reports of tonic immobility induced by scruff restraint (Lindsay & Russell, 2023; Lindsay, Holm et al., 2021; Vaillend & Chaussenot, 2017), vehicle‐treated *mdx* mice were not more sensitive than WT mice (Fig. 4*D*, *E*; *P* = 0.18). This suggests that repeated daily handling during treatment may have reduced the potency of this stressor, even in the absence of the more intense procedures typically used in the field, such as scruff restraint and oral gavage, possibly reflecting habituation to routine experimental contact.

To evaluate anxiety levels, we used the light–dark choice test and activity in the open field as physiological measures. Treatments did not alter time spent in the lit compartment (daily prednisolone: *P* = 0.94; weekly prednisolone: *P* = 0.88; daily vamorolone: *P *= 0.35; weekly vamorolone: *P* = 0.94), latency to enter (daily prednisolone: *P* = 1.00; weekly prednisolone: *P* = 1.00; daily vamorolone: *P *= 1.00; weekly vamorolone: *P* = 1.00) or number of light entries in the light–dark choice apparatus relative to *mdx* vehicle controls (Fig. 4*L*–*N*; Fig. A3*C*; daily prednisolone: *P* = 1.00; weekly prednisolone: *P* = 1.00; daily vamorolone: *P *= 1.00; weekly vamorolone: *P* = 1.00). Total distance travelled in the open field was also unaffected by treatment (Fig. 4*O*; daily prednisolone: *P* = 0.45; weekly prednisolone: *P* = 0.97; daily vamorolone: *P *= 0.94; weekly vamorolone: *P* = 1.00). Collectively, these data indicate that neither prednisolone nor vamorolone significantly modifies brain size or behavioural responses to stress and anxiety stimuli, suggesting that short‐term GC administration does not exacerbate or alleviate CNS physiological dysfunction in *mdx* mice.

### Daily glucocorticoid dosing drives extensive adrenal cortical atrophy

**Figure 5:**
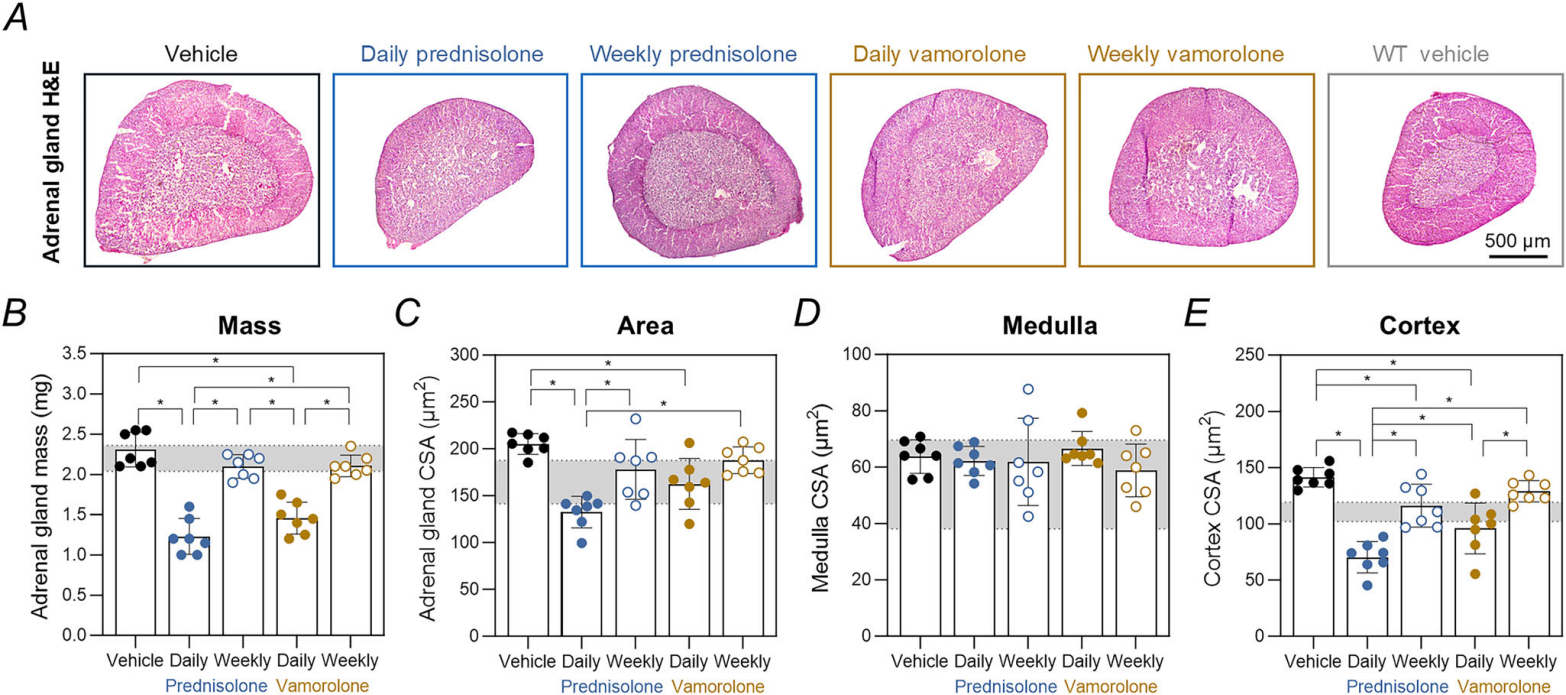
Daily glucocorticoid administration induces adrenal cortical atrophy, with a more pronounced effect following prednisolone compared with vamorolone *A*, representative haematoxylin and eosin (H&E)‐stained sections of adrenal glands (scale bar = 500 µm). *B*, adrenal gland mass. *C*, adrenal gland cross‐sectional area (CSA). *D*, adrenal medulla CSA. *E*, adrenal cortex CSA. Group differences were assessed using univariate ANOVA; *P* < 0.05. WT responses are shown in grey shading as ± SD. All other data are presented as mean ± SD; *n* = 7 mice per group.

**Figure A4:**
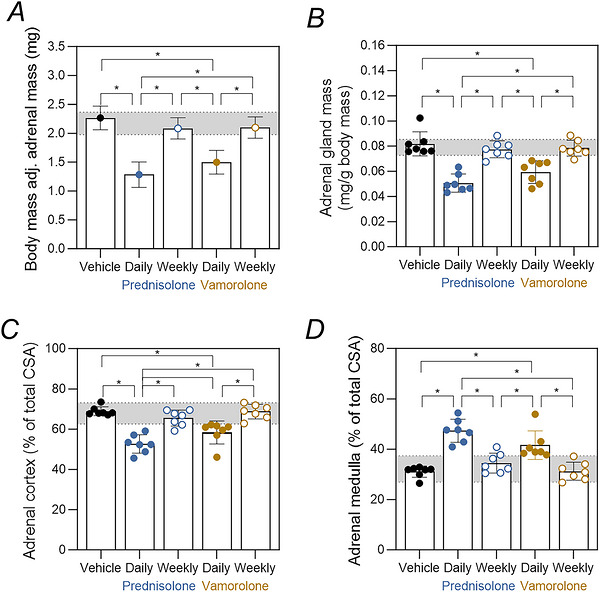
Overview of adrenal gland size and regional composition *A*, body‐weight‐adjusted adrenal gland mass across treatment groups. Data are estimated marginal means (±SD) derived from an ANCOVA model with body weight included as a covariate. *B*, adrenal gland mass expressed relative to body mass. *C*, adrenal gland cortex area expressed as a percentage of adrenal gland cross sectional area (CSA). *D*, adrenal gland medulla area expressed as a percentage of adrenal gland CSA. Note that the apparent increase in medullary percentage reflects cortical atrophy rather than genuine medullary enlargement, as absolute medulla CSA was unchanged across groups. Group differences were assessed using univariate ANOVA; *P* < 0.05. WT responses are shown in grey shading as ± SD. All other data, except where otherwise specified, are presented as mean ± SD; *n* = 7 mice per group.

Given the widespread impact of GCs on endocrine systems, and the central role of the HPA axis in stress regulation, we next examined adrenal morphology to assess whether treatment altered peripheral endocrine physiology, independent of behavioural or brain structural changes. Daily dosing of both prednisolone and vamorolone reduced overall adrenal mass (*P* = 0.000 and *P* = 0.000, respectively) and CSA (*P* = 0.000 and *P* = 0.010, respectively) relative to *mdx* vehicle controls, with daily administration resulting in lighter glands than weekly dosing (Fig. 5*A*–*C*; Fig. A4*A*, *B*; prednisolone: *P* = 0.000, vamorolone: *P* = 0.000). This effect was driven by selective atrophy of the adrenal cortex, as absolute medullary size remained unchanged across groups (Fig. 5*A*, *D*; Fig. A4*C*, *D*). Among all treatments, daily prednisolone caused the most pronounced cortical reduction, resulting in smaller cortical area than any other group (vehicle: *P* = 0.000; weekly prednisolone: *P* = 0.000; daily vamorolone: *P *= 0.028; weekly vamorolone: *P* = 0.000). However, weekly prednisolone (*P* = 0.032) and daily vamorolone (*P* = 0.000) also caused cortical atrophy, whereas weekly vamorolone preserved cortical size relative to vehicle controls (Fig. 5*A*, *E*; *P* = 0.61).

### Cardiovascular physiology remains largely stable across glucocorticoid treatments

**Figure 6:**
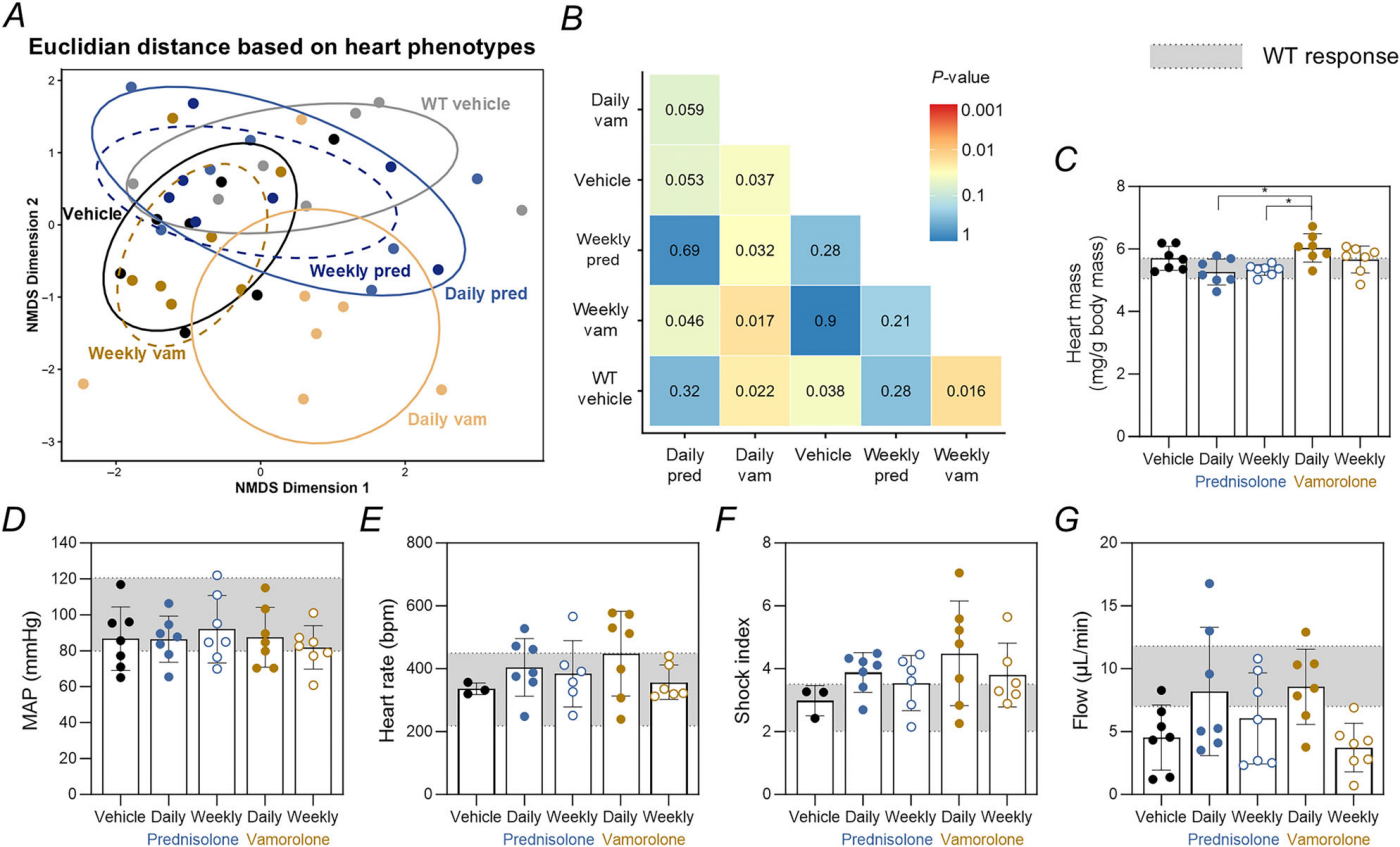
Striated muscle‐associated cardiovascular physiology remains largely stable across glucocorticoid treatments, with limited modulation restricted to daily vamorolone administration *A*, non‐metric multidimensional scaling (NMDS) ordination based on Euclidean distance illustrating multivariate striated muscle physiological profiles across experimental groups, including prednisolone (blue) and vamorolone (yellow) treatments, and WT (black) and *mdx* (grey) vehicle controls. Each point represents an individual animal, and proximity reflects similarity in integrated physiological profiles. *B*, heat map of pairwise *P*‐values for between‐group multivariate comparisons across all experimental groups. *C*, heart mass expressed relative to body mass; *D*, mean arterial pressure (MAP); *E*, heart rate; *F*, shock index (maximum heart rate/low systolic blood pressure; index of hypovolaemic shock); and *G*, tail blood flow. Multivariate group differences were assessed using Euclidean distance‐based PERMANOVA on z‐score standardised data, with dispersion tested by betadisper. Significant global effects were followed by pairwise multivariate comparisons, and outcome‐specific differences were assessed using univariate ANOVA with appropriate correction for multiple testing; *P* < 0.05. WT responses are shown in grey shading as ± SD. All other data are presented as mean ± SD; *n* = 3–7 mice per group.

**Figure A5:**
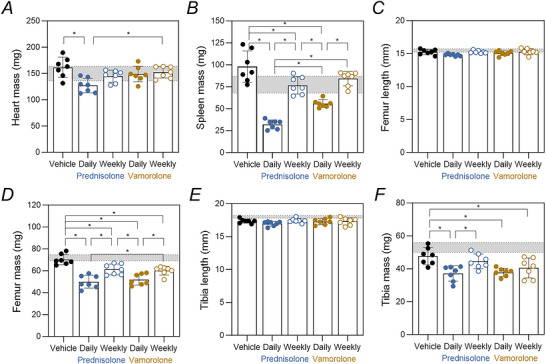
Overview of organ and bone size *A*, heart mass. *B*, spleen mass. *C*, femoral length. *D*, femoral mass. *E*, tibial length. *F*, tibial mass. Group differences were assessed using univariate ANOVA; *P* < 0.05. WT responses are shown in grey shading as ± SD. All other data are presented as mean ± SD; *n* = 7 mice per group.

To assess treatment‐dependent effects on cardiovascular physiology, we evaluated cardiac mass, haemodynamics and peripheral perfusion. NMDS was then performed to integrate outcomes from mean arterial pressure, heart rate, tail blood flow and heart mass into a single composite analysis. The NMDS showed that prednisolone, independent of dosing, did not alter overall heart physiology relative to *mdx* vehicle controls (daily prednisolone: *P* = 0.053; weekly prednisolone: *P* = 0.28), but daily vamorolone did have an effect (Fig. 6*A*, *B*; *P* = 0.037). Both daily (*P* = 0.32) and weekly (*P* = 0.28) prednisolone‐treated mice were also comparable to WT controls, unlike vamorolone‐ (daily vamorolone: *P* = 0.022; weekly vamorolone: *P* = 0.016) or vehicle‐treated *mdx* mice (Fig. 6*A*, *B*; *P* = 0.038). Daily vamorolone was associated with greater heart mass than prednisolone‐treated mice (daily prednisolone: *P* = 0.006; weekly prednisolone: *P* = 0.016), although no treatment differed from vehicle controls (Fig. 6*C*; Fig. A5*A*; daily prednisolone: *P* = 0.28; weekly prednisolone: *P* = 0.47; daily vamorolone: *P *= 0.57; weekly vamorolone: *P* = 1.00). Individually, treatment had no detectable effect on mean arterial pressure (daily prednisolone: *P* = 1.00; weekly prednisolone: *P* = 0.99; daily vamorolone: *P *= 1.00; weekly vamorolone: *P* = 0.99), heart rate (daily prednisolone: *P* = 0.95; weekly prednisolone: *P* = 0.99; daily vamorolone: *P *= 0.76; weekly vamorolone: *P* = 1.00), shock index (daily prednisolone: *P* = 0.80; weekly prednisolone: *P* = 0.97; daily vamorolone: *P *= 0.31; weekly vamorolone: *P* = 0.87) or tail blood flow relative to *mdx* vehicle controls (Fig. 6*D–G*; daily prednisolone: *P* = 0.32; weekly prednisolone: *P* = 0.95; daily vamorolone: *P *= 0.22; weekly vamorolone: *P* = 1.00), demonstrating the strength of integrated multivariate analysis in revealing subtle cardiovascular effects that single metrics alone cannot detect.

### Daily glucocorticoid administration disrupts systemic metabolic physiology and body composition

**Figure 7:**
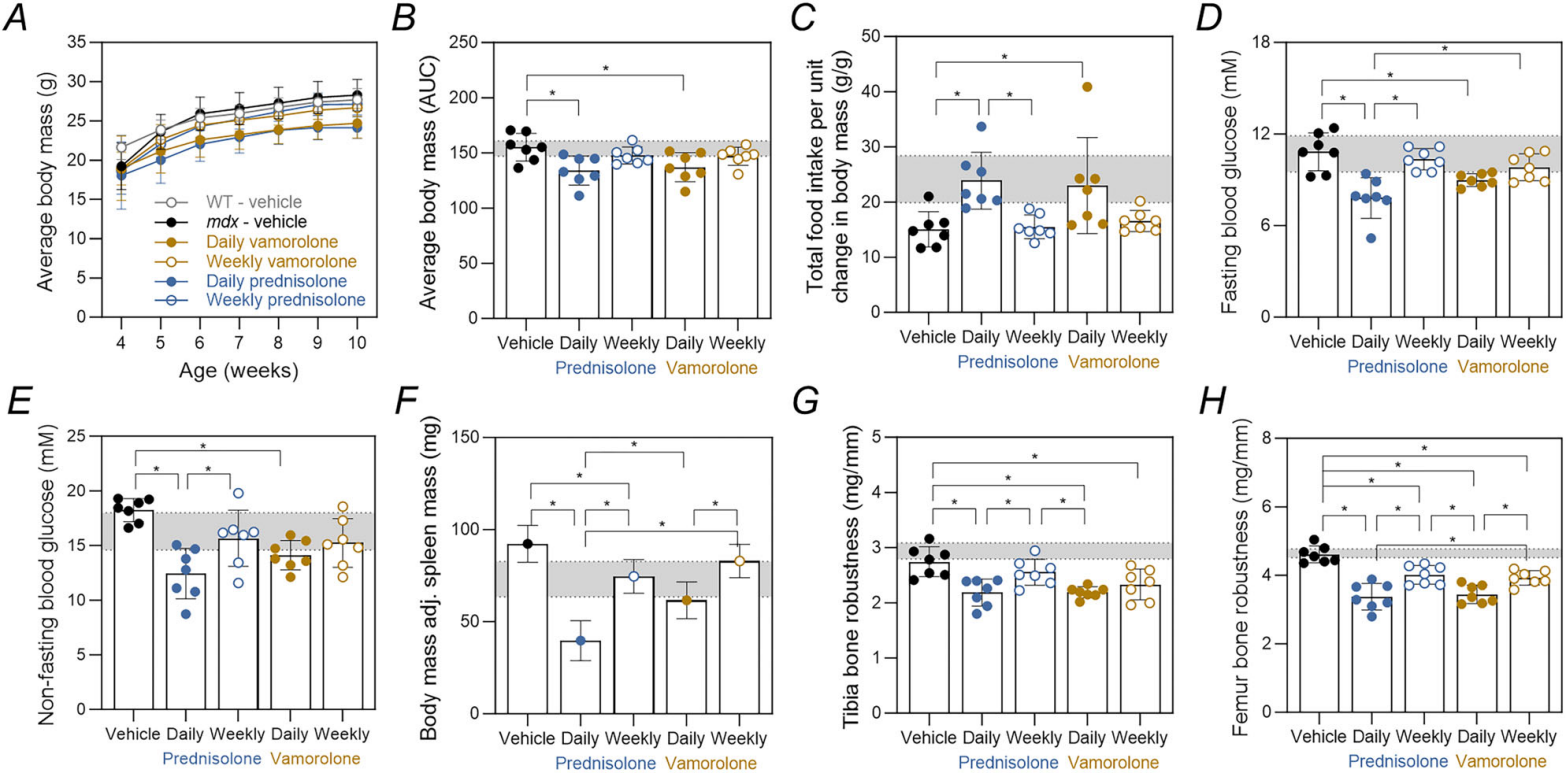
Daily glucocorticoids impair growth and body composition, causing hyperphagia, hypoglycaemia and reduced bone robustness, with spleen mass decreased only by daily prednisolone *A*, weekly body mass trajectories across the treatment period. *B*, body mass area under the curve (AUC). *C*, total food intake normalised to change in body mass over the treatment period. *D*, fasting blood glucose following 2 weeks of treatment. *E*, non‐fasting blood glucose following 6 weeks of treatment. *F*, body‐mass‐adjusted spleen mass across treatment groups. Data are estimated marginal means (±SD) derived from an ANCOVA model with body mass included as a covariate. *G*, tibial bone robustness (mass/length). *H*, femoral bone robustness (mass/length). Group differences were assessed using univariate ANOVA; *P* < 0.05. WT responses are shown in grey shading as ± SD. All other data, except where otherwise specified, are presented as mean ± SD; *n* = 7 mice per group.

We next assessed systemic metabolic function to determine how GC treatment regimens broadly impact whole‐body energy balance and organ physiology. Treating *mdx* mice during a juvenile growth window to model paediatric DMD, we observed that daily GCs impaired body mass growth (daily prednisolone: *P* = 0.008; daily vamorolone: *P *= 0.034), while paradoxically increasing food intake (daily prednisolone: *P* = 0.018; daily vamorolone: *P *= 0.042) relative to *mdx* vehicle controls (Fig. 7*A*–*C*). Both daily prednisolone and daily vamorolone reduced fasting (daily prednisolone: *P* = 0.004; daily vamorolone: *P *= 0.012) and non‐fasting (daily prednisolone: *P* = 0.000; daily vamorolone: *P *= 0.004) blood glucose levels relative to *mdx* vehicle controls, with no differences between these two daily treatments (Fig. 7*D*, *E*; *Fasting*: *P* = 0.74; *Non‐fasting*: *P* = 0.59). Daily dosing of GCs decreased spleen mass (daily prednisolone: *P* = 0.000; daily vamorolone: *P* = 0.000), although daily prednisolone caused a greater reduction than daily vamorolone (Fig. 7*F*; Fig. A5*B*; *P* = 0.0008). Weekly dosing of prednisolone (*P* = 0.012) but not vamorolone (*P* = 0.430) caused a reduction in spleen mass relative to *mdx* vehicle controls (Fig. 7*F*). Daily GCs reduced long bone robustness (bone mass per unit length, mg/mm) relative to *mdx* vehicle controls [*Tibia* (daily prednisolone: *P* = 0.0005; daily vamorolone: *P* = 0.0006); *femur* (daily prednisolone: *P* < 0.0001; daily vamorolone: *P* = 0.0000)], while weekly dosing produced milder but still measurable decreases in the femur [Fig. 7*G*, *H*; Fig. A5*C*–*F*; *Tibia* (daily prednisolone: *P* = 0.60; daily vamorolone: *P* = 0.016); *femur* (daily prednisolone: *P* = 0.002; daily vamorolone: *P* = 0.0004]). Overall, these findings demonstrate that daily GC exposure imposes broad metabolic and systemic effects compromising growth, energy balance, immune organ size and bone integrity, effects that are largely mitigated by weekly dosing.

## Discussion

We investigated how GC treatment affects striated muscle, systemic metabolism and CNS outcomes in juvenile *mdx* mice, comparing daily and weekly dosing of prednisolone and vamorolone over 6 weeks. Our results reveal that changes in striated muscle structure and function are mechanistically distinct and dosing regimen dependent, and that peripheral endocrine changes are largely uncoupled from CNS stress and anxiety responses. Weekly dosed vamorolone produced robust improvements in functional and *in vivo* strength relative to vehicle‐treated *mdx* mice, a notable finding, as previous studies of intermittent GC regimens (prednisolone and deflazacort) have not reported improvements in mass‐adjusted strength parameters (Quattrocelli et al., 2017). Daily prednisolone had minimal impact on torque generation despite reducing circulating CK and inducing muscle microstructural remodelling. System‐level NMDS analyses captured coordinated effects, highlighting compound‐ and regimen‐specific trade‐offs between muscle efficacy and systemic tolerability, including adrenal, metabolic and bone phenotypes, and revealing patterns not apparent from individual measures alone.

The relationship between striated muscle microstructure and functional performance is complex (Charles et al., 2022), and here we show that GC treatments can dissociate structural remodelling from strength outcomes in *mdx* mice, particularly under daily dosing regimens. Daily prednisolone reduced myofibre CSA and increased fibre density (number of fibres per unit area). Myofibres from vamorolone‐treated mice maintained the same fibre density as vehicle‐treated *mdx* mice despite smaller CSA. This remodelling pattern may reflect vamorolone's reduced catabolic effects compared to traditional GCs, consistent with its dissociative steroid mechanism (Crastin et al., 2025; Heier et al., 2019). Weekly dosing mitigated these structural effects across both compounds, consistent with previous reports that daily GC exposure triggers atrophy pathways and impairs voluntary muscle performance, whereas intermittent dosing avoids atrophy and preserves or improves muscle function (Quattrocelli et al., 2017). However, while prednisolone induced the greatest myofibre remodelling relative to vehicle‐treated *mdx* controls, these structural changes did not correlate with functional strength outcomes in either compound, highlighting how GC‐induced muscle fibre remodelling can occur independently of muscle performance in *mdx* mice. It remains possible that structure–function relationships may differ across muscles with distinct functional roles or loading patterns, an important consideration when interpreting single‑muscle histological analyses.

Limiting ongoing muscle damage is a critical goal in DMD, as repeated myofibre injury drives progressive weakness and disease progression (Duan et al., 2021). Daily prednisolone was the only regimen to reduce circulating CK, indicating meaningful protection against ongoing muscle damage. However, weekly dosing alone is not enough to mitigate damage given that weekly prednisolone was insufficient to reduce CK. Notably, these findings contrast with reports that vamorolone acts as a more potent membrane stabiliser than prednisolone and thus should theoretically produce even lower CK elevations (Heier et al., 2013). This discrepancy highlights the complexity of interpreting serum CK as a marker of dystrophic pathology, particularly in preclinical models. Circulating CK reflects sarcolemmal permeability and acute myofibre damage but does not reliably predict the degree of cumulative muscle damage, functional impairment or disease severity (Klein et al., 2017; TREAT‐NMD SOP MD_M.2.2.001), a limitation that is also recognised in clinical studies in humans. In *mdx* mice, CK levels are highly variable and can be influenced by factors such as muscle mass, activity levels and ongoing regeneration (c). Given the growth‑suppressive effects of daily GC administration and the inability to control for independent activity levels between treatment groups, CK levels in studies like this are therefore best interpreted as an indicator of acute muscle membrane instability rather than as a quantitative measure of overall disease severity.

While the primary physiological benefits of GC treatment in improving muscle function in DMD have been investigated (Baltgalvis et al., 2009; Bello et al., 2015; Guerron et al., 2010; McDonald et al., 2018; Morrison‐Nozik et al., 2015; Quattrocelli et al., 2017, 2019; Ricotti et al., 2013; Sali et al., 2012), much less is known about their neurocognitive and endocrine consequences. Using an integrative analysis, we confirm that *mdx* mice exhibit distinct stress and anxiety physiology relative to WT mice, yet GCs neither exacerbate nor alleviate these CNS dysfunctions. Limited GC penetration (Liu et al., 2024) or intrinsic GABAergic deficits (Vaillend & Chaussenot, 2017) in *mdx* mice may explain the relative insensitivity of these circuits. Supporting the latter, stress‐ and anxiety‐related behaviours remained unchanged despite clear adrenal cortical atrophy with daily dosing, demonstrating that central stress phenotypes are not simply driven by peripheral HPA‐axis output (Lindsay & Russell, 2023). Notably, adrenal cortical atrophy was most pronounced with daily prednisolone relative to vehicle‐treated *mdx* mice, whereas vamorolone, particularly when dosed weekly, largely spared adrenal structure, helping to reconcile heterogeneity in reported adrenal suppression across preclinical and clinical studies (Conklin et al., 2018; Dang et al., 2024; Guglieri et al., 2022; Heier et al., 2013; Liu et al., 2024). Together, these findings indicate that differential peripheral endocrine suppression does not translate into exacerbated CNS‐related phenotypes.

Cardiovascular physiology remained stable across treatments, reflecting minimal overt cardiac pathology in juvenile *mdx* mice (Adamo et al., 2010; Verhaart et al., 2012; Wasala et al., 2013). Daily vamorolone uniquely increased normalised heart mass relative to prednisolone‐treated mice and drove subtle NMDS shifts away from WT controls, despite no individual haemodynamic changes, raising the possibility of early pathological remodelling despite previously predicted cardioprotection via mineralocorticoid receptor antagonism (Heier et al., 2019). However, while juvenile *mdx* models best replicate early DMD skeletal muscle pathology, cardiovascular stability limits their utility for long‐term cardiac risk assessment, as echocardiographic defects emerge only after 10 months (Yucel et al., 2018).

Daily GC treatment imposed substantial systemic adverse effects in *mdx* mice, largely independent of muscle‐specific efficacy. Mice receiving daily GCs exhibited suppressed growth, reduced metabolic throughput, decreased spleen mass (prednisolone only) and compromised long bone robustness. Hyperphagia accompanied by low circulating glucose levels indicated inefficient substrate utilisation (Quattrocelli et al., 2019), consistent with a GC‐induced hypometabolic state characterised by suppressed growth (Quattrocelli et al., 2017), increased catabolism, reduced glucose production and impaired fasting‐induced glucose mobilisation. In contrast, weekly dosing, particularly of vamorolone, attenuated these systemic effects, highlighting dosing frequency as a key determinant of therapeutic index. These findings demonstrate that the systemic adverse effect profile is dose‐dependent and that careful optimisation of GC regimens can mitigate adverse outcomes while retaining potential therapeutic benefit.

This study was limited to juvenile *mdx* mice (4–10 weeks), which capture early muscle degeneration but not long‐term DMD progression, late cardiac pathology or chronic GC effects. The 6 week duration and single doses per regimen also restrict insights into sustained efficacy and dose optimisation, while modest CNS sample sizes may have obscured subtle neurobehavioural changes. Future studies should not only address these limitations but also elucidate the mechanistic basis for the observed structure–function dissociation and how optimised regimens reshape muscle regeneration at the cellular level.

## Additional information

## Competing interests

The authors have no conflicts of interest.

## Author contributions

The experiments were performed in the laboratory of A.L. G.M.: Conceptualisation; Methodology; Investigation; Data curation; Formal analysis; Interpretation of data; Visualisation; Funding acquisition; Project administration; Writing – original draft; Writing – review & editing. J.C.: Investigation (data acquisition); Visualisation; Writing – review & editing. E.vd.B.: Investigation (data acquisition); Visualisation; Writing – review & editing. D.M.: Interpretation of data; Writing – review & editing. A.L.: Conceptualisation; Writing – review & editing. All authors approved the final version of the manuscript and agree to be accountable for all aspects of the work. All authors qualify for authorship, and all those who qualify are listed.

## Funding

This work was supported by a Neurological Foundation First Fellowship awarded to G.M., and by a project grant from Neuromuscular Research New Zealand and the Richdale Charitable Trust. A.L. was supported by a Sir Charles Hercus Health Research Fellowship.

## Supporting information


Peer Review History

## Data Availability

All supporting data are included in the figures, with original raw data available from the corresponding author upon reasonable request.

## Appendix

**Table A1:** **Summary of statistical analyses for all outcome measures** For each variable, the table reports the statistical test used and the corresponding main effect *P* value. Where applicable, *post hoc* Tukey's HSD tests were performed following a significant ANOVA main effect. WT mice were included in all analyses.

Outcome measure	Statistical test	Main effect *P* value
**Main text data**		
Holding impulse (wire hang)	One‐way ANOVA, Tukey HSD	<0.0001
Time to fall (rotarod)	One‐way ANOVA	0.15
Specific tetanic torque (anterior muscles)	One‐way ANOVA, Tukey HSD	<0.0001
Contraction (anterior muscles)	One‐way ANOVA, Tukey HSD	0.004
Relaxation (anterior crural)	One‐way ANOVA	0.11
Hydroxyproline	One‐way ANOVA, Tukey HSD	<0.0001
Diaphragm diameter	One‐way ANOVA, Tukey HSD	<0.0001
Creatine kinase	One‐way ANOVA, Tukey HSD	<0.0001
Centronucleated fibres	One‐way ANOVA, Tukey HSD	<0.0001
Myofibre CSA	One‐way ANOVA, Tukey HSD	<0.0001
No. of myofibres/mm^2^	One‐way ANOVA, Tukey HSD	0.001
Movement time (scruff)	One‐way ANOVA, Tukey HSD	0.01
Freezing (scruff)	Two‐way ANOVA, Tukey HSD	Group × time: 0.01 Group: 0.003 Time: 0.30 Mouse: <0.0001
Centre time (scruff)	One‐way ANOVA	0.13
Movement time (tube)	One‐way ANOVA, Tukey HSD	<0.0001
Freezing (tube)	Two‐way ANOVA, Tukey HSD	Group × time: 0.71 Group: <0.0001 Time: <0.0001 Mouse: 0.0003
Centre time (tube)	One‐way ANOVA, Tukey HSD	0.001
Time in lit compartment	One‐way ANOVA	0.055
Light entry count	Kruskal–Wallis	0.52
Total distance travelled	One‐way ANOVA, Tukey HSD	0.010
Adrenal gland mass	One‐way ANOVA, Tukey HSD	<0.0001
Adrenal gland CSA	One‐way ANOVA, Tukey HSD	<0.0001
Medulla CSA	One‐way ANOVA	0.33
Cortex CSA	One‐way ANOVA, Tukey HSD	<0.0001
Heart mass [relative to body mass (BM)]	One‐way ANOVA, Tukey HSD	0.004
Mean arterial pressure	One‐way ANOVA, Tukey HSD	0.43
Heart rate	One‐way ANOVA, Tukey HSD	0.40
Shock index	One‐way ANOVA, Tukey HSD	0.066
Tail blood flow	One‐way ANOVA, Tukey HSD	0.011
Average body mass (AUC)	One‐way ANOVA, Tukey HSD	0.002
Food intake	One‐way ANOVA, Tukey HSD	<0.0001
Fasting blood glucose	One‐way ANOVA, Tukey HSD	0.002
Non‐fasting blood glucose	One‐way ANOVA, Tukey HSD	<0.0001
Body mass adju. spleen mass	One‐way ANOVA, Tukey HSD	<0.0001
Tibia bone robustness	One‐way ANOVA, Tukey HSD	<0.0001
Femur bone robustness	One‐way ANOVA, Tukey HSD	<0.0001
**Supplementary data**		
Maximum isometric torque (anterior muscles)	One‐way ANOVA, Tukey HSD	<0.0001
Specific tetanic torque (posterior muscles)	One‐way ANOVA, Tukey HSD	<0.0001
Maximum isometric torque (posterior muscles)	One‐way ANOVA, Tukey HSD	<0.0001
Contraction (posterior muscles)	One‐way ANOVA, Tukey HSD	0.004
Relaxation (posterior muscles)	One‐way ANOVA, Tukey HSD	0.001
TA mass (relative to BM)	One‐way ANOVA, Tukey HSD	<0.0001
EDL mass (relative to BM)	One‐way ANOVA, Tukey HSD	0.027
Gastrocnemius mass (relative to BM)	One‐way ANOVA, Tukey HSD	0.009
Soleus mass (relative to BM)	One‐way ANOVA, Tukey HSD	0.002
TA mass	One‐way ANOVA, Tukey HSD	<0.0001
EDL mass	One‐way ANOVA, Tukey HSD	0.009
Gastrocnemius mass	One‐way ANOVA, Tukey HSD	0.002
Soleus mass	One‐way ANOVA, Tukey HSD	0.005
Brain mass (relative to BM)	One‐way ANOVA, Tukey HSD	<0.0001
Brain mass	One‐way ANOVA	0.051
Light latency	Kruskal–Wallis	0.44
Body mass adj. adrenal mass	One‐way ANOVA, Tukey HSD	<0.0001
Adrenal gland mass (relative to BM)	One‐way ANOVA, Tukey HSD	<0.0001
Adrena cortex (% CSA)	One‐way ANOVA, Tukey HSD	<0.0001
Adrena medulla (% CSA)	One‐way ANOVA, Tukey HSD	<0.0001
Heart mass	One‐way ANOVA, Tukey HSD	0.003
Spleen mass	One‐way ANOVA, Tukey HSD	<0.0001
Femur length	One‐way ANOVA, Tukey HSD	0.01
Femur mass	One‐way ANOVA, Tukey HSD	<0.0001
Tibia length	One‐way ANOVA, Tukey HSD	0.001
Tibia mass	One‐way ANOVA, Tukey HSD	<0.0001
