# Supplementary material for: Regimen‐dependent glucocorticoid effects improve muscle performance without altering CNS physiology in mdx mice
Source: J Physiol. 2026 May 28;604(14):5984–6008. doi: 10.1113/JP291378 (PMC13370735; doi:10.1113/JP291378)
Supplement: Supplementary file 1 — Peer Review History [file TJP-604-5984-s001.pdf]

The Journal of Physiology

Regimen-dependent glucocorticoid effects improve muscle performance without altering CNS physiology in *mdx* mice

Gretel S Major, Jiayi Chen, Eva Salina van den Berg, Deirdre L Merry, and Angus Lindsay
DOI: 10.1113/JP291378

Corresponding author(s): Gretel Major (gretel.major@canterbury.ac.nz)

The referees have opted to remain anonymous.

Review Timeline:

Submission Date:	15-Mar-2026
Editorial Decision:	30-Mar-2026
Revision Received:	13-Apr-2026
Accepted:	12-May-2026

Senior Editor: Paul Greenhaff

Reviewing Editor: Kevin Murach

Transaction Report:

(Note: With the exception of the correction of typographical or spelling errors that could be a source of ambiguity, letters and reports are not edited. Depending on transfer agreements, referee reports obtained elsewhere may or may not be included in this compilation. Referee reports are anonymous unless the Referee chooses to sign their reports.)

Re: JP-RP-2026-291378 "Regimen-dependent glucocorticoid effects improve muscle performance without altering CNS physiology in mdx mice" by Gretel S Major, Jiayi Chen, Eva van den Berg, Deirdre L Merry, and Angus Lindsay

Dear Dr Major,

Thank you for submitting your manuscript to The Journal of Physiology. It has been assessed by a Reviewing Editor and by 2 expert referees and we are pleased to tell you that it is potentially acceptable for publication following satisfactory major revision.

Please advise your co-authors of this decision as soon as possible.

The referee reports are copied at the end of this email.

Please address all the points raised and incorporate all requested revisions or explain in your Response to Referees why a change has not been made. We hope you will find the comments helpful and that you will be able to return your revised manuscript within 2 months. If your article is NOT for a Special Issue, you may have 9 months to revise. If you require an extension, please contact journal staff: jp@physoc.org. Please note that this letter does not constitute a guarantee for acceptance of your revised manuscript.

Your revised manuscript should be submitted online using the link in your Author Tasks: Link Not Available. This link is accessible via your account as Corresponding Author; it is not available to your co-authors. If this presents a problem, please contact journal staff (jp@physoc.org). Image files from the previous version are retained on the system. Please ensure you replace or remove any files that are being revised.

If you do not wish to submit a revised version of your manuscript, you must inform our journal staff (jp@physoc.org) or reply to this email to request withdrawal. Please note that a manuscript must be formally withdrawn from the peer review process at one journal before it may be submitted to another journal.

TRANSPARENT PEER REVIEW POLICY: To improve the transparency of its peer review process, The Journal of Physiology publishes online as supporting information the peer review history of all articles accepted for publication. Readers will have access to decision letters, including Editors' comments and referee reports, for each version of the manuscript, as well as any author responses to peer review comments. Referees can decide whether or not they wish to be named on the peer review history document.

ABSTRACT FIGURES: Authors are expected to use The Journal's premium BioRender account to create/redraw their Abstract Figures. Information on how to access this account is here: https://physoc.onlinelibrary.wiley.com/journal/14697793/biorender-access.

This will enable Authors to create and download high-resolution figures. If authors have used the free BioRender service, they can use the instructions provided in the link above to download a high-resolution version suitable for publication.

The link provided should only be used for the purposes of this submission. Authors will be charged for figures created on this account if they are not related to this manuscript submission.

LANGUAGE EDITING AND SUPPORT FOR PUBLICATION: If you would like help with English language editing, or other article preparation support, Wiley Editing Services offers expert help, including English Language Editing, as well as translation, manuscript formatting, and figure formatting at www.wileyauthors.com/eeo/preparation. You can also find resources for Preparing Your Article for general guidance about writing and preparing your manuscript at www.wileyauthors.com/eeo/prepresources.

RIGOUR AND REPRODUCIBILITY: We recommend authors consult The Journal's Rigour and Reproducibility requirements to ensure that their manuscript is suitable for publication: https://physoc.onlinelibrary.wiley.com/pb-assets/hub-assets/physoc/documents/TJP-Rigour-and-Reproducibility-Requirements.pdf

REVISION CHECKLIST:

Check that your Methods section conforms to journal policy: https://jp.msubmit.net/cgi-bin/main.plex?form_type=display_requirements#methods.

Check that data presented conforms to the statistics policy: https://jp.msubmit.net/cgi-bin/main.plex?form_type=display_requirements#statistics.

Upload a full Response to Referees file. To create your 'Response to Referees': copy all the reports, including any comments from the Senior and Reviewing Editors, into a Microsoft Word, or similar, file and respond to each point, using font or background colour to distinguish comments and responses and upload as the required file type.

Please upload two versions of your manuscript text: one with all relevant changes highlighted and one clean version with no changes tracked. The manuscript file should include all tables and figure legends, but each figure/graph should be uploaded as separate, high-resolution files.

You may also upload:

- 'Potential Cover Art' for consideration as the issue's cover image
- Appropriate Supporting Information (Video, audio or data set: see https://jp.msubmit.net/cgi-bin/main.plex?form_type=display_requirements#supp).

We look forward to receiving your revised submission.

If you have any queries, please reply to this email and we will be pleased to advise.

Yours sincerely,

Paul Greenhaff
Senior Editor
The Journal of Physiology

EDITOR COMMENTS

Reviewing Editor:

Your work has been evaluated by two experts in the field. Both agreed that the word is novel and impactful. Their comments primarily pertain to clarification and interpretation. Your work will be further considered with satisfactory revision.

Senior Editor:

Comments for Authors to ensure the paper complies with the Statistics Policy (Required):
I have raised this in the SE response to the authors.

Comments to the Author:
Thank you for the manuscript submission to The Journal of Physiology. It has been considered by a reviewing editor and two expert reviewers. Overall there were positive comments from both reviews and the Reviewing Editor, such that the study was deemed to be potentially important and influential. However, several issues were raised by both reviewers which require consideration and further clarification by the authors. Furthermore, details of anaesthetic protocols and monitoring, and humane killing need to be included in the Methods section. Thank you for including mean and individual values, but please note The Journal requires the use of SD, not SEM throughout. In addition, please use exact P values, not < and > throughout. We look forward to receiving the revised manuscript and response to the reviewers.

REFEREE COMMENTS

Referee #1:

Major et al. investigated the effects of daily or weekly glucocorticoid treatment, using prednisolone or vamorolone, on C57BL/10 mdx mouse muscle pathology, whole-body metabolism, and CNS physiology. Their results show that weekly vamorolone dosing improved muscle strength in mdx mice, demonstrated by better 4-limb hang test results and in vivo specific torque production, while also reducing some adverse side effects - though this is attributed to the weekly glucocorticoid dosing rather than the specific type of glucocorticoid. Neither glucocorticoid nor treatment strategy worsened or accelerated the anxiety observed in vehicle-treated mdx mice. Overall, this is an important and comprehensive study that adopts a holistic approach when investigating the effects of glucocorticoid treatment on mdx mice. I do have a few concerns that should be addressed.

1. The authors should discuss how the doses for each glucocorticoid were chosen.

2. One of the main findings was that weekly vamorolone treatment enhanced specific torque production when normalized to TA and EDL mass. The authors should present both absolute torque values and the actual sizes of TA and EDL, along with other muscles shown in supplementary figure 1. Is the increase in specific torque a result of normalization?

3. The CK results are interesting and raise questions about the utility of serum CK as a marker in dystrophic pathology. The

authors may want to discuss this.

4. In section 3.2, the authors state: "Importantly, structural parameters such as fibre size, density, and central nucleation did not correlate with functional performance measures, rotarod performance, four-limb wire holding impulses, and in vivo torque, in mdx mice (Figure S2), indicating that muscle architecture alone does not predict functional outcomes." It is difficult to accept this statement since the structural parameters were only measured in the TA muscle. Aside from the in vivo torque measures, how representative is the TA muscle of these measures?

5. I do not see any methods relating to the cardiovascular physiology outcomes.

Referee #2:

With this manuscript Major et al. aim to compare daily and weekly prednisolone (5mg/kg/day) and vamorolone (30 mg/kg/day) in juvenile mdx mice.

The authors should be complimented for a well-written manuscript addressing a relevant topic.

I have some minor points that I would like clarified, and one larger concern regarding the data comparisons and interpretations, which I find to be very important, however, I believe this can be addressed by a minor revision.

Specific input by section:

Introduction: The introduction is very well written and sets the scene as it should.

Materials and methods:

- There is no information on the total number of mice used. The figure text states n=6-7 per group, however, I find it relevant to know if these are the same 6-7 mice for all analyses. Further, the text states there are 5 groups, however, I assume the WT group is a sixth group?

- Creatine kinase: In humans CK, is a good marker for the presence of damage, however, it cannot predict the degree of damage. Is this different in mice? If so, the data should be interpreted with caution.

- Statistics: This section was difficult to read. I would like it clarified which tests are used and when. Further, it would be interesting to know how many missing data you had, and that therefore had to be imputed. While the figure text states the use of either only ANOVA or ANOVA and Kruskal-Wallis, it is not clear which data are analysed with which test. Further, it is unclear if the WT-group is part of the statistical comparisons,

Results:

- I cannot find the results from the ANOVA. While you have written that you only do post hoc testing if there is a significant main effect, I would like to see the results of the main effect.

- Your aim is to compare two different GC treatments and daily vs. weekly dosing. The only comparisons I find relevant in this regard are:

1) Vehicle vs. all

2) Daily vs. weekly for prednisolone

3) Daily vs. weekly for vamorolone

4) Daily prednisolone vs. daily vamorolone

5) Weekly prednisolone vs. weekly varmorolone

- I recognize that the Tukey's post hoc test gives you all comparisons, thus showing the results are statistically valid, however, I don't agree that you can interpret e.g. daily prednisolone vs. weekly vamorolone, as this compares both treatment type and dose of treatment.

- Showing the results from the WT group is inconsistent. Is there a reason for this?

- Section 3.2 half-way down you write "...with weekly dosing producing larger fibres than daily dosing..." Can you conclude

this? Fibre size does not differ from vehicle, so are they producing larger fibres, or is the fibre size diminished in the mice receivings daily dosing?

- Section 3.3, last line you write "... with prednisolone showing superior efficacy" . You show lower CK in daily prednisolone compared to weekly vamorolone, but is this due to prednisolone or the dosing regimen? I agree it is superior to vehicle, but I would be cautious in saying it is superior, without specifying the comparator.

- Figure 4 C: Can you show the individual data points, like you do for the other outcomes?

Discussion

All of my comments to this section relate to the between group comparisons, where, as noted above, I think you should be cautious. See examples below:

- Weekly-dosed vamorolone was not superior to daily vamorolone, therefor I question your statement that it produced the "greatest gains"

- The following section starting with "in contrast". I don't see the contrast, further, I don't follow the statement. Daily prednisolone does not differ from any of the other groups (if you are referring to figure 2E)

- Section two "...GC treatment appears to dissociate structural remodelling...", this seems only evident with daily dosing as I read the figures. Weekly dosing shows no difference from vehicle

- The text "...prednisolone induced more pronounced myofibre remodelling relative to vamorolone.." I interpret this differently. The difference is only evident when comparing daily to weekly dosing. Thus, how can you determine whether this is due to the GC type or the timing of the doses.

- Page two: "Notably, adrenal atrophy was most pronounced with prednisolone..." As above, how can you dissociate drug effect from timing?

END OF COMMENTS

Reference Number: JP-RP-2026-291378
Title: Regimen-dependent glucocorticoid effects improve muscle performance without altering CNS physiology in *mdx* mice

Authors: Gretel S. Major, Jiayi Chen, Eva van den Berg, Deirdre L. Merry and Angus Lindsay

Reviewer's comments: Black
Author's response: Blue
Text Insertions: Green (page and line numbers are all in mark-up mode in main document)
Text Deletions: Red (page and line numbers are all in mark-up mode in main document)

Response to comments:
We thank the editors and reviewers for their supportive comments and constructive recommendations which have helped improve the clarity and significance of our manuscript. We have included a point-by-point response to the comments below. Line numbers correspond to the revised manuscript containing full markup.

EDITOR COMMENTS

Reviewing Editor:

Your work has been evaluated by two experts in the field. Both agreed that the word is novel and impactful. Their comments primarily pertain to clarification and interpretation. Your work will be further considered with satisfactory revision.

Senior Editor:

Comments for Authors to ensure the paper complies with the Statistics Policy (Required): I have raised this in the SE response to the authors.

Comments to the Author:

Thank you for the manuscript submission to The Journal of Physiology. It has been considered by a reviewing editor and two expert reviewers. Overall there were positive comments from both reviews and the Reviewing Editor, such that the study was deemed to be potentially important and influential. However, several issues were raised by both reviewers which require consideration and further clarification by the authors. Furthermore, details of anaesthetic protocols and monitoring, and humane killing need to be included in the Methods section. Thank you for including mean and individual values, but please note The Journal requires the use of SD, not SEM throughout. In addition, please use exact P values, not < and > throughout. We look forward to receiving the revised manuscript and response to the reviewers.

Information on anaesthesia, physiological monitoring, and humane killing has now been added to the Methods section under *Anaesthesia and euthanasia*, in accordance with ARRIVE guidelines. Data are now presented as standard deviation throughout the manuscript, and exact P values are reported consistently. Where inequality symbols (≤, ≥) were previously used to summarise multiple P values, these have now been replaced with explicitly defined values in parentheses (e.g., **Line 328-330**). The only exception is where P values are genuinely below 0.0001, which are reported as $P < 0.0001$ to ensure clarity and accuracy.

Line 241-248 Mice were anaesthetised for *in vivo* skeletal muscle function assessment using isoflurane delivered in 95% oxygen. Anaesthesia was induced in an induction chamber (5% isoflurane) and maintained via a nose cone at 1-3% isoflurane for the duration of the experiment. Body temperature was maintained at 37°C using a thermostatically controlled heating pad. Depth of anaesthesia was assessed and monitored throughout by absence of the pedal withdrawal reflex. Upon completion of the experimental protocol, mice remained under deep isoflurane anaesthesia and terminal blood was collected by intracardiac puncture. Animals were then humanely killed by cervical dislocation while fully unconscious.

REFEREE COMMENTS

Referee #1:

Major et al. investigated the effects of daily or weekly glucocorticoid treatment, using prednisolone or vamorolone, on C57BL/10 mdx mouse muscle pathology, whole-body metabolism, and CNS physiology. Their results show that weekly vamorolone dosing improved muscle strength in mdx mice, demonstrated by better 4-limb hang test results and in vivo specific torque production, while also reducing some adverse side effects - though this is attributed to the weekly glucocorticoid dosing rather than the specific type of glucocorticoid. Neither glucocorticoid nor treatment strategy worsened or accelerated the anxiety observed in vehicle-treated mdx mice. Overall, this is an important and comprehensive study that adopts a holistic approach when investigating the effects of glucocorticoid treatment on mdx mice. I do have a few concerns that should be addressed.

1. The authors should discuss how the doses for each glucocorticoid were chosen.

We thank the reviewer for this helpful comment. We have now expanded the description of dose selection in the Methods section. The original statement indicating that the doses were consistent with previous preclinical studies has been revised to provide a clearer rationale for the chosen prednisolone and vamorolone doses, including their basis in established dosing paradigms and, where applicable, prior intermittent glucocorticoid studies.

Line 140-147 These dosages are consistent with previous preclinical studies evaluating vamorolone (Heier *et al*.. 2013, 2019; McCormack *et al.,* 2023; Liu *et al.,* 2024) and prednisolone (Liu *et al., 2024; Kourakis* et al., 2024).

Line 142-149 The oral prednisolone dose (5 mg/kg/d) was selected based on established dosing paradigms shown to modify muscle pathology and function in mdx mice (Liu et al., 2024; Kourakis et al., 2024). The intermittent glucocorticoid regimen was informed by prior studies demonstrating beneficial effects using intraperitoneal administration of prednisolone at 1mg/kg/d (Quattrocelli et al., 2017, 2019) (Quattrocelli et al., 2017, 2019). Intermittent dosing has not previously been examined for vamorolone; therefore, the 30 mg/kg/d oral dose was chosen based on studies demonstrating efficacy with daily administration in dystrophic mice (Heier et al., 2013, 2019; McCormack et al., 2023; Liu et al., 2024).

2. One of the main findings was that weekly vamorolone treatment enhanced specific torque production when normalized to TA and EDL mass. The authors should present both absolute torque values and the actual sizes of TA and EDL, along with other muscles shown in supplementary figure 1. Is the increase in specific torque a result of normalization?

We thank the reviewer for the suggestion to include both absolute and normalised values and agree this is useful to the readership. In response, we have now added the absolute torque values and the individual muscle masses (TA, EDL, soleus, gastroc) to **Figure S1**, as requested. These data show that weekly vamorolone treatment results in significantly greater absolute torque production in the anterior crural muscles compared with daily prednisolone and daily vamorolone treatment. However, we also observe differences in body and muscle size across treatment groups, consistent with the known growth-suppressive effects of glucocorticoids. For this reason, normalisation of torque to muscle mass is necessary to accurately assess intrinsic muscle performance independent of treatment-related differences in muscle size. We therefore retain specific torque as the primary outcome measure, while providing the absolute values and muscle sizes to allow full transparency and interpretation.

Line 865-875 Figure S1. Evaluation of absolute isometric strength of anterior crural muscles, posterior leg muscle isometric strength, contractile properties, and muscle mass. (A) Absolute *in vivo* isometric muscle strength of the anterior crural muscles. (B) *In vivo* isometric muscle strength expressed as specific tetanic torque normalised to gastrocnemius (gas) and soleus (sol) muscle mass. (C) Absolute *in vivo* isometric muscle strength of the posterior leg muscles. (D) Contraction kinetics of posterior leg muscles expressed as maximal rate of torque development. (E) Relaxation kinetics of posterior leg muscles expressed as maximal rate of torque relaxation. (F) Tibialis anterior mass expressed relative to body mass. (G) EDL muscle mass expressed relative to body mass. (H) Gastrocnemius mass expressed relative to body mass. (I) Soleus mass expressed relative to body mass. (J) Absolute tibialis anterior mass. (K) Absolute EDL mass. (L) Absolute gastrocnemius mass. (M) Absolute soleus mass. Group differences were assessed using univariate ANOVA; $P < 0.05$. WT responses are shown in grey shading as ± SD. All other data are presented as mean ± SD; n = 7 mice per group.

3. The CK results are interesting and raise questions about the utility of serum CK as a marker in dystrophic pathology. The authors may want to discuss this.

We agree with the reviewer that further discussion of CK as a marker of dystrophic pathology would be an important discussion point, and have expanded the text accordingly. Specifically, we now discuss that CK reflects acute sarcolemmal instability rather than cumulative damage or functional status and that CK values in *mdx* mice are highly variable. This interpretation is supported by prior studies demonstrating poor correlation between CK and functional impairment (Klein et al., 2017) and by the TREAT-NMD guidance on serum CK measurement in dystrophic mouse models.

Line 614-623 This discrepancy highlights the complexity of interpreting serum CK as a marker of dystrophic pathology, particularly in preclinical models. Circulating CK reflects sarcolemmal permeability and acute myofibre damage but does not reliably predict the degree of cumulative muscle damage, functional impairment, or disease severity (Klein et al., 2017; TREAT-NMD SOP MD_M.2.2.001), a limitation that is also recognised in clinical studies in humans. In *mdx* mice, CK levels are highly variable and can be influenced by factors such as muscle mass, activity levels, and ongoing regeneration (TREAT-NMD SOP MD_M.2.2.001). Given the growth-suppressive effects of daily glucocorticoid administration and the inability to control for independent activity levels between treatment groups, CK levels in studies like this are therefore best interpreted as an indicator of acute muscle membrane instability rather than as a quantitative measure of overall disease severity.

Line 624-625 Although CK reduction did not translate to short-term gains, this represents a disease-modifying effect that may confer longer-term function benefit beyond the scope of this study.

4. In section 3.2, the authors state: "Importantly, structural parameters such as fibre size, density, and central nucleation did not correlate with functional performance measures, rotarod performance, four-limb wire holding impulses, and in vivo torque, in mdx mice (Figure S2), indicating that muscle architecture alone does not predict functional outcomes." It is difficult to accept this statement since the structural parameters were only measured in the TA muscle. Aside from the in vivo torque measures, how representative is the TA muscle of these measures?

We thank the reviewer for raising this point. We agree that structural parameters were measured only in the TA and therefore cannot be assumed to be fully representative of all muscle groups contributing to whole-body functional tasks such as the rotarod and four-limb wire hang. Our intention was not to imply global structure-function dissociation across all muscles, but rather to examine whether commonly used histopathological indices in the TA predict functional outcomes within this cohort. Importantly, the TA (together with the EDL) is the primary contributor to anterior crural torque, and thus represents an anatomically matched comparison for the *in vivo* torque measurements. We have revised the text to clarify that the lack of correlation should be interpreted in a muscle- and task-specific context and to acknowledge the limitation of extrapolating TA histology to whole-body performance measures.

Line 400-405 Importantly, structural parameters measured in the tibialis anterior, including fibre size, density, and central nucleation, did not correlate with anterior crural *in vivo* torque or whole-body functional performance measures (rotarod performance and four-limb wire holding impulse) in *mdx* mice (**Figure S2**). These findings indicate that tibialis anterior muscle architecture alone is insufficient to predict functional outcomes across anatomically distinct and multi-muscle tasks.

Line 605-607 It remains possible that structure-function relationships may differ across muscles with distinct functional roles or loading patterns, an important consideration when interpreting single-muscle histological analyses.

5. I do not see any methods relating to the cardiovascular physiology outcomes.

Methods relating to cardiovascular physiology outcomes are described under **Section 2.5.4**, “Tube-restraint stress test with haemodynamic measures” (**line 202-212**). This section details the assessment of cardiovascular parameters using the CODA non-invasive blood pressure system, including measurement of mean arterial pressure, heart rate, tail blood volume, and shock index during a standardised restraint protocol. Because acquisition of these cardiovascular parameters requires placement in a plexiglass restraint within the CODA system, all measurements were obtained under standardised restraint-induced stress conditions. To aid in easier detection of these methods we have modified the wording.

Line 202-204 2.5.4 Tube-restraint stress test with cardiovascular haemodynamic measures: The haemodynamic response to stress was measured using a 5 min tube-restraint stressor with concurrent evaluation of cardiovascular haemodynamic parameters.

Referee #2:

With this manuscript Major et al. aim to compare daily and weekly prednisolone (5mg/kg/day) and vamorolone (30 mg/kg/day) in juvenile mdx mice. The authors should be complimented for a well-written manuscript addressing a relevant topic. I have some minor points that I would like clarified, and one larger concern regarding the data comparisons and interpretations, which I find to be very important, however, I believe this can be addressed by a minor revision.

Specific input by section:

Introduction: The introduction is very well written and sets the scene as it should.

Materials and methods

1. There is no information on the total number of mice used. The figure text states n=6-7 per group, however, I find it relevant to know if these are the same 6-7 mice for all analyses. Further, the text states there are 5 groups, however, I assume the WT group is a sixth group?

We thank the reviewer for raising this point and have now clarified the total number of animals used and the group structure in the Methods section. A total of n = 42 mice were used, comprising five mdx treatment groups and a sixth wild-type (WT) vehicle control group. Group sizes were n = 7 per group. The same cohort of mice was used across behavioural, *in vivo* functional, biochemical, and histological analyses. These details are now stated explicitly in Sections 2.2 and 2.3.

Line 119 A total of n = 42 mice were used in this study.

Line 127-135 Six experimental groups were evaluated: five *mdx* treatment groups – 1) cherry syrup vehicle, 2) 5 mg/kg prednisolone daily, 3) 5 mg/kg prednisolone weekly, 4) 30 mg/kg vamorolone daily and 5) 30 mg/kg vamorolone weekly – and a sixth group of WT mice receiving the cherry syrup vehicle as a healthy control. Group sizes were n = 7 mice per group and the same animals were used across longitudinal, behavioural, *in vivo* functional, biochemical, and histological analyses. For brain mass and behavioural outcomes, final analyses were performed with *n* = 6 mice in the *mdx* vehicle control group following post-mortem identification of hydrocephalus at endpoint dissection.

2. Creatine kinase: In humans CK, is a good marker for the presence of damage, however, it cannot predict the degree of damage. Is this different in mice? If so, the data should be interpreted with caution.

We appreciate this important point and agree with the reviewer. We have clarified in the Discussion that the limitations of CK interpretation in humans also apply to *mdx* mice, where CK reflects acute membrane damage but does not reliably predict disease severity or functional outcomes. We now explicitly note that CK levels in *mdx* mice are influenced by factors such as activity, muscle mass, and regeneration and therefore require cautious interpretation, consistent with both clinical understanding and established preclinical guidance (TREAT-NMD SOP MD_M.2.2.001; Klein et al., 2017).

Line 614-623 This discrepancy highlights the complexity of interpreting serum CK as a marker of dystrophic pathology, particularly in preclinical models. Circulating CK reflects sarcolemmal permeability and acute myofibre damage but does not reliably predict the degree of cumulative muscle damage, functional impairment, or disease severity (Klein et al., 2017; TREAT-NMD SOP MD_M.2.2.001), a limitation that is also recognised in clinical studies in humans. In *mdx* mice, CK levels

are highly variable and can be influenced by factors such as muscle mass, activity levels, and ongoing regeneration (TREAT-NMD SOP MD_M.2.2.001). Given the growth-suppressive effects of daily glucocorticoid administration and the inability to control for independent activity levels between treatment groups, CK levels in studies like this are therefore best interpreted as an indicator of acute muscle membrane instability rather than as a quantitative measure of overall disease severity.

Line 624-625 Although CK reduction did not translate to short-term gains, this represents a disease-modifying effect that may confer longer-term function benefit beyond the scope of this study.

3. Statistics: This section was difficult to read. I would like it clarified which tests are used and when. Further, it would be interesting to know how many missing data you had, and that therefore had to be imputed. While the figure text states the use of either only ANOVA or ANOVA and Kruskal-Wallis, it is not clear which data are analysed with which test. Further, it is unclear if the WT-group is part of the statistical comparisons,

We thank the reviewer for this constructive feedback. The Statistics section has been revised substantially to clarify these points raised. A new supplementary **Table S1** now details every outcome measure alongside the statistical test used, and the main effect P values. We have also explicitly clarified that WT mice were included in all statistical comparisons. In addition, we now report the number and nature of missing data points and specify that imputation was applied only for multivariate PERMANOVA analyses.

Line 301-315 Heart rate measurements could not be obtained for six mice (four *mdx* vehicle controls, one weekly vamorolone, and one weekly prednisolone), resulting in missing data for this variable only. In addition, one mouse in the daily vamorolone group refused the four-limb wire hang test and was excluded from that specific analysis. Missing data were imputed solely for the purpose of multivariate PERMANOVA following standardisation of variables. No imputation was applied to any univariate analyses. Excluding the mice with missing values entirely yielded comparable PERMANOVA results.

For analysis of individual outcome measures, group comparisons were performed using one-way ANOVA for single-factor designs, two-way ANOVA for repeated-measures behavioural outcomes (binned freezing behaviour), or Kruskal-Wallis test when normality assumption was not met and could not be corrected (light-dark apparatus), with a P value of < 0.05 considered statistically significant. Where relevant, post hoc Tukey's HSD tests were used for multiple comparisons following ANOVA. Details of the statistical test used and main effect P values for all outcome measures are detailed in **Table S1.** WT mice were included in all statistical tests; however results are described with emphasis on treatment related differences within *mdx* mice and comparisons indicating restoration towards WT levels.

Line 914-918 Table S1. Summary of statistical analyses for all outcome measures. For each variable, the table reports the statistical test used, the corresponding main effect P value. Where applicable, post hoc Tukey's HSD tests were performed following a significant ANOVA main effect. WT mice were included in all analyses.

**Outcome measure**	**Statistical test**	**Main effect P value**
Main text data		
Holding impulse (wire hang)	1- way ANOVA, Tukey HSD	< 0.0001
Time to fall (rotarod)	1- way ANOVA	0.15
Specific tetanic torque (anterior muscles)	1- way ANOVA, Tukey HSD	< 0.0001
Contraction (anterior muscles)	1- way ANOVA, Tukey HSD	0.004
Relaxation (anterior crural)	1- way ANOVA	0.11

Hydroxyproline	1- way ANOVA, Tukey HSD	< 0.0001
Diaphragm diameter	1- way ANOVA, Tukey HSD	< 0.0001
Creatine kinase	1- way ANOVA, Tukey HSD	< 0.0001
Centronucleated fibres	1- way ANOVA, Tukey HSD	< 0.0001
Myofibre CSA	1- way ANOVA, Tukey HSD	< 0.0001
# myofibres/mm^2	1- way ANOVA, Tukey HSD	0.001
Movement time (scruff)	1- way ANOVA, Tukey HSD	0.01
Freezing (scruff)	2-way ANOVA, Tukey HSD	Group x time: 0.01<br>Group: 0.003<br>Time: 0.30<br>Mouse: < 0.0001
Centre time (scruff)	1- way ANOVA	0.13
Movement time (tube)	1- way ANOVA, Tukey HSD	< 0.0001
Freezing (tube)	2-way ANOVA, Tukey HSD	Group x time: 0.71<br>Group: < 0.0001<br>Time: < 0.0001<br>Mouse: 0.0003
Centre time (tube)	1- way ANOVA, Tukey HSD	0.001
Time in lit compartment	1- way ANOVA	0.055
Light entry count	Kruskal-Wallis	0.52
Total distance travelled	1- way ANOVA, Tukey HSD	0.010
Adrenal gland mass	1- way ANOVA, Tukey HSD	< 0.0001
Adrenal gland CSA	1- way ANOVA, Tukey HSD	< 0.0001
Medulla CSA	1- way ANOVA	0.33
Cortex CSA	1- way ANOVA, Tukey HSD	< 0.0001
Heart mass (relative to BM)	1- way ANOVA, Tukey HSD	0.004
MAP	1- way ANOVA, Tukey HSD	0.43
Heart rate	1- way ANOVA, Tukey HSD	0.40
Shock index	1- way ANOVA, Tukey HSD	0.066
Tail blood flow	1- way ANOVA, Tukey HSD	0.011
Average body mass (AUC)	1- way ANOVA, Tukey HSD	0.002
Food intake	1- way ANOVA, Tukey HSD	< 0.0001
Fasting blood glucose	1- way ANOVA, Tukey HSD	0.002
Non-fasting blood glucose	1- way ANOVA, Tukey HSD	< 0.0001
Body mass adju. spleen mass	1- way ANOVA, Tukey HSD	< 0.0001
Tibia bone robustness	1- way ANOVA, Tukey HSD	< 0.0001
Femur bone robustness	1- way ANOVA, Tukey HSD	< 0.0001
Supplementary data		
Max isometric torque (anterior muscles)	1- way ANOVA, Tukey HSD	< 0.0001
Specific tetanic torque (posterior muscles)	1- way ANOVA, Tukey HSD	< 0.0001
Max isometric torque (posterior muscles)	1- way ANOVA, Tukey HSD	< 0.0001
Contraction (posterior muscles)	1- way ANOVA, Tukey HSD	0.004
Relaxation (posterior muscles)	1- way ANOVA, Tukey HSD	0.001
TA mass (relative to BM)	1- way ANOVA, Tukey HSD	< 0.0001
EDL mass (relative to BM)	1- way ANOVA, Tukey HSD	0.027
Gastrocnemius mass (relative to BM)	1- way ANOVA, Tukey HSD	0.009
Soleus mass (relative to BM)	1- way ANOVA, Tukey HSD	0.002
TA mass	1- way ANOVA, Tukey HSD	< 0.0001
EDL mass	1- way ANOVA, Tukey HSD	0.009
Gastrocnemius mass	1- way ANOVA, Tukey HSD	0.002

Soleus mass	1- way ANOVA, Tukey HSD	0.005
Brain mass (relative to BM)	1- way ANOVA, Tukey HSD	< 0.0001
Brain mass	1- way ANOVA	0.051
Light latency	Kruskal-Wallis	0.44
Body mass adj. adrenal mass	1- way ANOVA, Tukey HSD	< 0.0001
Adrenal gland mass (relative to BM)	1- way ANOVA, Tukey HSD	< 0.0001
Adrena cortex (% CSA)	1- way ANOVA, Tukey HSD	< 0.0001
Adrena medulla (% CSA)	1- way ANOVA, Tukey HSD	< 0.0001
Heart mass	1- way ANOVA, Tukey HSD	0.003
Spleen mass	1- way ANOVA, Tukey HSD	< 0.0001
Femur length	1- way ANOVA, Tukey HSD	0.01
Femur mass	1- way ANOVA, Tukey HSD	< 0.0001
Tibia length	1- way ANOVA, Tukey HSD	0.001
Tibia mass	1- way ANOVA, Tukey HSD	< 0.0001

Results:

4. I cannot find the results from the ANOVA. While you have written that you only do post hoc testing if there is a significant main effect, I would like to see the results of the main effect.

We agree that inclusion of ANOVA main effects improves transparency. We now report the main effects and corresponding P values for all ANOVA analyses in **Table S1** (above).

5. Your aim is to compare two different GC treatments and daily vs. weekly dosing. The only comparisons I find relevant in this regard are:

   1) Vehicle vs. all

   2) Daily vs. weekly for prednisolone

   3) Daily vs. weekly for vamorolone

   4) Daily prednisolone vs. daily vamorolone

   5) Weekly prednisolone vs. weekly varmorolone

   I recognize that the Tukey's post hoc test gives you all comparisons, thus showing the results are statistically valid, however, I don't agree that you can interpret e.g. daily prednisolone vs. weekly vamorolone, as this compares both treatment type and dose of treatment.

This issue was carefully considered during experimental design and statistical planning. Our primary aim was to evaluate the net physiological effects of clinically relevant glucocorticoid treatment regimens, rather than to formally partition variance attributable independently to compound identity and dosing frequency.

Prednisolone and vamorolone differ substantially in pharmacology, potency, and required dose, and therefore a two-way ANOVA framework would assume factor independence and dose equivalence that are not biologically justified in this context. Applying such a model could give a misleading

impression of mechanistic separation between drug and dosing effects. For this reason, we elected to use a global one-way ANOVA approach with post hoc Tukey testing, which allows unbiased identification of outcome-specific differences across all treatment regimens.

While Tukey's post hoc testing yields statistical comparisons between all groups, our interpretation is intentionally constrained to biologically relevant contrasts, including: (i) vehicle versus treatment effects, (ii) within-compound daily versus weekly dosing effects, and (iii) compound-specific comparisons at matched dosing frequency. Comparisons that span both drug identity and dosing schedule (e.g. daily prednisolone versus weekly vamorolone) are reported for completeness and transparency, but are not interpreted as isolating independent effects of compound or dose.

Accordingly, conclusions throughout the manuscript have been reframed in terms of regimen-level effects rather than drug-intrinsic or dosing-intrinsic causality (examples provided in following comments), an approach we believe is statistically appropriate and biologically conservative given the study design.

6. Showing the results from the WT group is inconsistent. Is there a reason for this?

WT mice were included in all statistical analyses and are displayed consistently across figures as SD ranges. In the Results, however, we focused discussion on treatment-related differences within *mdx* mice and on comparisons indicating restoration towards WT levels, rather than reiterating expected disease–control differences across all outcome measures. This approach was adopted to maintain clarity and avoid unnecessary repetition. We have added details of this to the text.

Line 312-315 Details of the statistical test used and main effect P values for all outcome measures are detailed in Table S1. WT mice were included in all statistical tests; however results are described with emphasis on treatment related differences within mdx mice and comparisons indicating restoration towards WT levels.

7. Section 3.2 half-way down you write "...with weekly dosing producing larger fibres than daily dosing..." Can you conclude this? Fibre size does not differ from vehicle, so are they producing larger fibres, or is the fibre size diminished in the mice receivings daily dosing?

We thank the reviewer for suggesting this important clarification and agree with their interpretation. We have revised the text to avoid implying that weekly dosing induces fibre hypertrophy.

Line 394-396 In prednisolone-treated mice, fibre size was also influenced by dosing frequency (P = 0.002), reflecting reduced fibre cross-sectional area with daily dosing **(Figure 3A,C).**

8. Section 3.3, last line you write "... with prednisolone showing superior efficacy" . You show lower CK in daily prednisolone compared to weekly vamorolone, but is this due to prednisolone or the dosing regimen? I agree it is superior to vehicle, but I would be cautious in saying it is superior, without specifying the comparator.

We have revised the results text to avoid implying drug-intrinsic superiority of prednisolone over vamorolone. The revised wording now clarifies that reduced CK levels were observed specifically with daily prednisolone administration.

Line 391-385 Weekly vamorolone-treated mice had higher CK than both daily prednisolone- (P = 0.0003) and daily vamorolone-treated mice (P = 0.024), demonstrating that daily GC administration is

required to mitigate muscle damage, with prednisolone showing superior efficacy and that daily prednisolone was the only regimen associated with a significant reduction in circulating CK relative to *mdx* vehicle controls.

9. Figure 4 C: Can you show the individual data points, like you do for the other outcomes?

We thank the reviewer for this suggestion. Figure 4C presents body-mass–adjusted brain mass, which was analysed using estimated marginal means derived from a statistical model rather than plotted directly from raw individual measurements. This approach was necessary to account for the non-linear scaling relationship between brain mass and body size, as well as treatment-related differences in somatic growth. Glucocorticoid treatment markedly alters body mass, and simple ratio-based normalisation can therefore introduce artefactual differences that reflect growth suppression rather than CNS-specific effects. Accordingly, brain mass was adjusted using a covariate-based model to account for body mass differences across groups. As this yields model-estimated adjusted values rather than directly observed measurements, individual data points cannot be meaningfully displayed. A description of this is found in **line 464-466.**

Discussion:

10. All of my comments to this section relate to the between group comparisons, where, as noted above, I think you should be cautious. See examples below:
    - Weekly-dosed vamorolone was not superior to daily vamorolone, therefor I question your statement that it produced the "greatest gains"
    - The following section starting with "in contrast". I don't see the contrast, further, I don't follow the statement. Daily prednisolone does not differ from any of the other groups (if you are referring to figure 2E)
    - Section two "...GC treatment appears to dissociate structural remodelling...", this seems only evident with daily dosing as I read the figures. Weekly dosing shows no difference from vehicle
    - The text "...prednisolone induced more pronounced myofibre remodelling relative to vamorolone.." I interpret this differently. The difference is only evident when comparing daily to weekly dosing. Thus, how can you determine whether this is due to the GC type or the timing of the doses.
    - Page two: "Notably, adrenal atrophy was most pronounced with prednisolone..." As above, how can you dissociate drug effect from timing?

We thank the reviewer for their detailed reading of the manuscript and for the thoughtful interpretation of the data. In response, we have revised the Results and Discussion to adopt a more cautious and precise approach to between-group comparisons. Language implying superiority or suggesting that drug effects can be dissociated from dosing regimen has been removed or rephrased where this is not directly supported by the data.

Dear Dr Major,

Re: JP-RP-2026-291378R1 "**Regimen-dependent glucocorticoid effects improve muscle performance without altering CNS physiology in *mdx* mice**" by Gretel S Major, Jiayi Chen, Eva Salina van den Berg, Deirdre L Merry, and Angus Lindsay

We are pleased to tell you that your paper has been accepted for publication in The Journal of Physiology.

The last Word (or similar) version of the manuscript provided will be used by the Production Editor to prepare your proof. When this is ready you will receive an email containing a link to Wiley's Online Proofing System. The proof should be thoroughly checked and corrected as promptly as possible.

Please note that as the journal does not permit supplementary figures and tables, we will include this material in an Appendix, within the article. Please check your proofs very carefully in due course.

Authors should note that it is too late at this point to offer corrections prior to proofreading. Major corrections at proof stage, such as changes to figures, will be referred to the Editors for approval before they can be incorporated. Only minor changes, such as to style and consistency, should be made at proof stage. Changes that need to be made after proof stage will usually require a formal correction notice.

All queries at proof stage should be sent to: TJP@wiley.com.

Following copyediting and typesetting the accepted version will be published online.

Yours sincerely,

Paul Greenhaff
Senior Editor
The Journal of Physiology

IMPORTANT POINTS TO NOTE FOLLOWING ACCEPTANCE OF YOUR PAPER:

• IMPORTANT NOTICE ABOUT OPEN ACCESS: To assist authors whose funding agencies mandate immediate public access to published research findings, The Journal of Physiology allows authors to pay an Open Access (OA) fee to have their papers made freely available immediately on publication.

The Corresponding Author will receive an email from Wiley with details on how to register or log in to Wiley Authors where you will be able to place an order.

• You can check if your funder or institution has a Wiley Open Access Account here:
https://authors.wiley.com/author-resources/Journal-Authors/open-access/author-compliance-tool.html

• TRANSPARENT PEER REVIEW POLICY: To improve the transparency of its peer review process, The Journal of Physiology publishes online as supporting information the peer review history of all articles accepted for publication. Readers will have access to decision letters, including Editors' comments and referee reports, for each version of the manuscript as well as any author responses to peer review comments. Referees can decide whether or not they wish to be named on the peer review history document.

• You can help your research get the attention it deserves! Check out Wiley's free Promotion Guide for best-practice recommendations for promoting your work at: www.wileyauthors.com/eeo/guide. You can learn more about Wiley Editing Services which offers professional video, design, and writing services to create shareable video abstracts, infographics, conference posters, lay summaries, and research news stories for your research at: www.wileyauthors.com/eeo/promotion.

• If you would like to receive our 'Research Roundup', a monthly newsletter highlighting the cutting-edge research published in The Physiological Society's family of journals (The Journal of Physiology, Experimental Physiology, Physiological Reports, The Journal of Nutritional Physiology and The Journal of Precision Medicine: Health and Disease), please click this link, fill in your name and email address and select 'Research Roundup':
https://www.physoc.org/journals-and-media/membernews

EDITOR COMMENTS

Reviewing Editor:

Comments to the Author (Required):
Thank you for your responsiveness to the reviewers. Both are now satisfied. Thank you for submitting your work to the Journal of Physiology.

Senior Editor:

Comments to the Author:
Thank you for the revised manuscript and rebuttal document, which have been considered by the same reviewing editor and reviewers that considered the original manuscript submission. I am happy to convey that all were of the opinion that the authors have addressed the previous review comments adequately and that the revised manuscript has been improved. The manuscript is now deemed acceptable for publication. Congratulations and thank you for supporting The Journal of Physiology.

REFEREE COMMENTS

Referee #1:

The authors have addressed all of my concerns.

Referee #2:

I appreciate you thorough responses to all my comments, and the text edits were relevant. I have no further comments.
